# Cwp19 Is a Novel Lytic Transglycosylase Involved in Stationary-Phase Autolysis Resulting in Toxin Release in *Clostridium difficile*

**DOI:** 10.1128/mBio.00648-18

**Published:** 2018-06-12

**Authors:** Sandra Wydau-Dematteis, Imane El Meouche, Pascal Courtin, Audrey Hamiot, René Lai-Kuen, Bruno Saubaméa, François Fenaille, Marie-José Butel, Jean-Louis Pons, Bruno Dupuy, Marie-Pierre Chapot-Chartier, Johann Peltier

**Affiliations:** a EA 4065, CRP2, DHU Risks in pregnancy, Faculté de Pharmacie de Paris, Université Paris Descartes, Université Sorbonne Paris Cité, Paris, France; b Laboratoire GRAM (EA 2656 IFR 23 IHURBM), Université de Rouen, Rouen, France; c Micalis Institute, INRA, AgroParisTech, Université Paris-Saclay, Jouy-en-Josas, France; d Laboratoire Pathogenèse des Bactéries Anaérobies, Institut Pasteur, Paris, France; e Cellular and Molecular Imaging Platform, CRP2, UMS 3612 CNRS, US25 INSERM, Faculté de Pharmacie de Paris, Université Paris Descartes, Sorbonne Paris Cité, France; f Université Paris Diderot, Université Sorbonne Paris Cité, Paris, France; g CEA, Institut Joliot, Service De Pharmacologie et d’Immunoanalyse, UMR0496, Laboratoire d’Etude du Métabolisme des Médicaments, MetaboHUB, Université Paris Saclay, Paris, France; KUMC

**Keywords:** *Clostridium difficile*, autolysins, lytic transglycosylase, peptidoglycan, surface proteins, toxins

## Abstract

Clostridium difficile is the major etiologic agent of antibiotic-associated intestinal disease. Pathogenesis of C. difficile is mainly attributed to the production and secretion of toxins A and B. Unlike most clostridial toxins, toxins A and B have no signal peptide, and they are therefore secreted by unusual mechanisms involving the holin-like TcdE protein and/or autolysis. In this study, we characterized the cell surface protein Cwp19, a newly identified peptidoglycan-degrading enzyme containing a novel catalytic domain. We purified a recombinant His_6_-tagged Cwp19 protein and showed that it has lytic transglycosylase activity. Moreover, we observed that Cwp19 is involved in cell autolysis and that a C. difficile *cwp19* mutant exhibited delayed autolysis in stationary phase compared to the wild type when bacteria were grown in brain heart infusion (BHI) medium. Wild-type cell autolysis is correlated to strong alterations of cell wall thickness and integrity and to release of cytoplasmic material. Furthermore, we demonstrated that toxins were released into the extracellular medium as a result of Cwp19-induced autolysis when cells were grown in BHI medium. In contrast, Cwp19 did not induce autolysis or toxin release when cells were grown in tryptone-yeast extract (TY) medium. These data provide evidence for the first time that TcdE and bacteriolysis are coexisting mechanisms for toxin release, with their relative contributions *in vitro* depending on growth conditions. Thus, Cwp19 is an important surface protein involved in autolysis of vegetative cells of C. difficile that mediates the release of the toxins from the cell cytosol in response to specific environment conditions.

## IMPORTANCE

Clostridium difficile-associated disease is mainly known as a health care-associated infection. It represents the most problematic hospital-acquired infection in North America and Europe and exerts significant economic pressure on health care systems. Virulent strains of C. difficile generally produce two toxins that have been identified as the major virulence factors. The mechanism for release of these toxins from bacterial cells is not yet fully understood but is thought to be partly mediated by bacteriolysis. Here we identify a novel peptidoglycan hydrolase in C. difficile, Cwp19, exhibiting lytic transglycosylase activity. We show that Cwp19 contributes to C. difficile cell autolysis in the stationary phase and, consequently, to toxin release, most probably as a response to environmental conditions such as nutritional signals. These data highlight that Cwp19 constitutes a promising target for the development of new preventive and curative strategies.

## INTRODUCTION

Clostridium difficile, a Gram-positive spore-forming bacterium, is the major cause of intestinal disease associated with antibiotic therapy (1). C. difficile infection (CDI) is a serious risk within health care environments and is increasingly recognized as a community-associated disease (2, 3). Outbreaks leading to increased morbidity and mortality have been associated with highly virulent strains such as BI/NAP1/027 (4). CDI can result in a spectrum of symptoms, ranging from mild diarrhea to life-threatening pseudomembranous colitis and colon perforation (5). The major virulence factors mediating these symptoms are toxins A and B (TcdA and TcdB), which alter the actin cytoskeleton of intestinal epithelial cells through glucosylation of Rho family proteins, leading to a severe inflammatory response and inducing the symptoms associated with CDI (6).

TcdA and TcdB belong to the family of large clostridial glucosylating toxins (LCGTs) (7). A feature of LCGTs is that they are secreted without an identifiable secretion signal. The *tcdA* and *tcdB* genes lie within a 19.6-kb pathogenicity locus (PaLoc), which also includes three additional genes designated *tcdR*, *tcdC*, and *tcdE*. TcdR is an alternative sigma factor that initiates transcription from the *tcdA*, *tcdB*, and *tcdR* promoters (8). TcdC is thought to be an anti-sigma factor for TcdR (9), but its function is still a subject of debate (10). TcdE is a protein with structural features similar to those found in holins of bacteriophages (11) that has been shown to be involved in toxin release from C. difficile cells (11–13). In addition, a link between toxin release and peptidoglycan (PG) hydrolase (PGH)-induced bacteriolysis has also been suggested (14).

PG is the major component of the bacterial cell wall that helps to maintain cell shape and confers resistance to internal osmotic pressure (15, 16). PG is composed of linear glycan strands made of alternating *N*-acetylglucosamine (NAG) and *N*-acetylmuramic acid (NAM) and cross-linked by short peptides. PG is constantly remodeled during bacterial growth and division to allow the incorporation of new PG subunits and to accommodate changes in cell shape. This remodeling requires PG-degrading enzymes capable of cleaving certain covalent bonds of the cell wall PG (17). PGHs represent a key class of enzymes responsible for hydrolyzing PG bonds. Four different PGH activities are defined according to the chemical bond cleaved inside the PG molecule as follows: *N*-acetylmuramyl-l-alanine amidase, endopeptidase, *N*-acetylglucosaminidase, and *N*-acetylmuramidase (18). Another common family of PG-degrading enzymes is that of the lytic transglycosylases (LTs). LTs cleave the same bonds as muramidases, but they are not hydrolases since they use the C-6 hydroxyl of NAM rather than water as a nucleophile in the cleavage reaction, yielding a product ending with 1,6-anhydroNAM (19). PG-degrading enzymes contribute to various physiological functions such as cell wall expansion, cell wall turnover, and daughter cell separation after division (17, 20). PG-degrading enzymes also include bacterial autolysins, whose activity may lead to autolysis and consequently to release of the intracellular content under specific conditions, such as exposure to a stress (21). Finely tuned regulation of the PG-degrading enzyme activity is therefore essential for bacterial survival (22). In addition to their functions in the bacterial physiology, many autolysins of Gram-positive bacteria are implicated in pathogenicity by mediating bacterial adherence or by releasing virulence factors and can be required for full virulence in animal models. These include Atl and Sle1 in Staphylococcus aureus (23, 24); AtlE in S. epidermidis (25); LytA, LytB, and LytC in Streptococcus pneumoniae (26–29); and IspC, Auto, and p60 in Listeria monocytogenes (30–32).

Very little is known about the PG-degrading enzymes in C. difficile. Acd, a PGH with *N*-acetylglucosaminidase activity, and two proteins exhibiting *N*-acetylmuramyl-l-alanine amidase activity, the CD11 and CDG enzymes, have been identified, but their physiological functions have not yet been investigated (33, 34). In addition, SleC, a spore cortex lytic enzyme with LT activity, has been shown to be essential for germination of C. difficile spores (35, 36). Furthermore, the putative endopeptidase SpoIIQ was recently shown to be required for normal spore engulfment, although its catalytic domain seems to be dispensable for this function (37, 38).

In this work, we identified a novel PG-degrading enzyme, Cwp19, with an uncharacterized catalytic domain and demonstrated that Cwp19 functions as an LT. Inactivation of *cwp19* decreased Triton X-100-induced autolysis. Moreover, cell autolysis following entry into the stationary phase was remarkably delayed in the *cwp19* mutant compared to the wild-type strain when bacteria were cultivated in brain heart infusion (BHI) medium. Cell autolysis was accompanied by a reduction of cell wall thickness and alterations of cell wall integrity. A drastic reduction in toxin secretion was also observed in the strain lacking Cwp19, suggesting that toxin release was primarily mediated by Cwp19-induced autolysis in cells grown in BHI medium. However, the growth medium appeared to significantly influence Cwp19 activity since Cwp19 did not induce stationary-phase autolysis or toxin release in cells grown in tryptone-yeast extract (TY) medium. This suggests the contribution of the TcdE-dependent mechanism of toxin secretion.

## RESULTS

### Cell wall protein Cwp19 is a peptidoglycanase.

**FIG 1:**
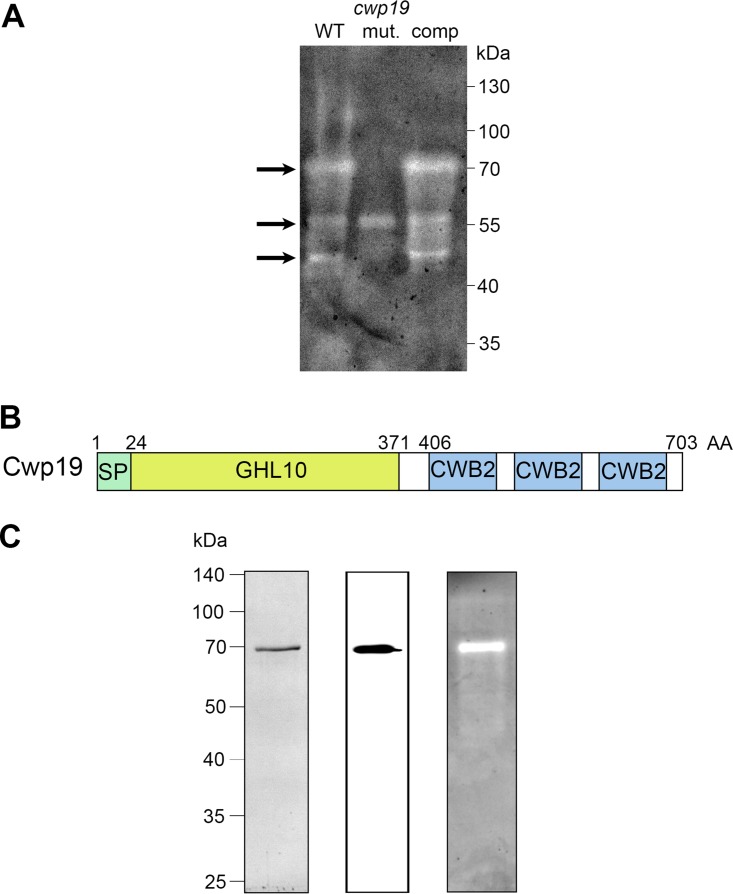
Cwp19 is a PG-degrading enzyme. (A) Detection of bacteriolytic activities in surface protein extracts of C. difficile 630 Δ*erm* (WT), *cwp19* mutant (mut.), and complemented *cwp19* mutant (comp) strains by zymogram. The gel contained 0.2% (wt/vol) M. lysodeikticus cells as a substrate. Surface proteins were extracted from cells grown to the exponential phase in BHI medium. Arrows indicate the positions of the bacteriolytic bands. (B) Schematic representation of Cwp19. The relevant characteristics are a signal peptide (SP), a putative catalytic domain (GHL10), and three copies of a cell wall binding motif termed CWB2. (C) Analysis of purified His_6_-tagged Cwp19 by SDS-PAGE (left panel), Western blotting performed with anti-His antibody (middle panel), and zymogram assay (right panel).

PG-degrading enzymes are classically detected by zymogram assays, in which the substrate (whole bacteria or bacterial PG) is incorporated into the resolving gel of SDS-PAGE. This allows the assay to reveal PG-degrading activity of proteins by clear lytic zones in the gel. The peptidoglycanase activity of surface proteins extracted from C. difficile 630Δ*erm* was examined by zymogram with Micrococcus lysodeikticus cells as the substrate. A major lytic band with a molecular weight of 70 kDa and two minor lytic bands with molecular weights of around 55 and 50 kDa were detected (Fig. 1A). Using *in silico* analysis of catalytic domains of PG-degrading enzymes (18), 32 putative peptidoglycanase-encoding genes were found in the genome of C. difficile 630 (see Table S1 in the supplemental material). Several of them (*acd*, *cwp6*, *CD0961*, *CD1035*, *CD1036*, *CD1108*, and *CD2903*) encode proteins with a theoretical molecular weight of close to 70 kDa, making the prediction of the protein responsible for the major hydrolytic activity detected by zymogram assay difficult. To identify the 70-kDa PG-degrading enzyme, surface proteins of C. difficile 630Δ*erm* were separated by SDS-PAGE and proteins with a size around 70 kDa were excised from the gel and digested with trypsin and tryptic peptides were analyzed by liquid chromatography-tandem mass spectrometry (LC-MS/MS). Three different proteins were thus identified: the well-characterized cysteine protease Cwp84 (39, 40), the surface layer (S-layer) precursor protein SlpA (41), and the cell wall protein Cwp19. Surprisingly, none of the peptidoglycanases predicted from the *in silico* analysis were found among the identified proteins.

10.1128/mBio.00648-18.6 TABLE S1  List of predicted PG-degrading enzymes identified in C. difficile 630. Download TABLE S1, DOCX file, 0.03 MB. Copyright © 2018 Wydau-Dematteis et al. 2018 Wydau-Dematteis et al. This content is distributed under the terms of the Creative Commons Attribution 4.0 International license.

Cwp19 is a putative surface-associated protein of 703 amino acids with a predicted molecular weight of 77.8 kDa and a pI of 8.99. This protein has a putative 24-amino acid signal sequence, a C-terminal domain containing three tandem copies of the cell wall binding motif CWB2 (Pfam04122), and a putative N-terminal catalytic domain belonging to the glycosyl hydrolase-like 10 family (GHL10) (Pfam02638) (Fig. 1B). Cwp19 is conserved in every genome available for C. difficile. Moreover, the GHL10 domain is widely distributed in bacterial species (Table S2). At the time of our study, we found in the Pfam database 1,642 GHL10 domain-containing proteins from 871 organisms (42). These proteins are frequently found in members of the Gram-positive phyla *Firmicutes* and *Actinobacteria* as well as in Gram-negative bacteria, including members of the phyla *Bacteroidetes*, *Cyanobacteria*, and *Proteobacteria*.

10.1128/mBio.00648-18.7 TABLE S2  Phylogenetic distribution of GHL10 domain. Download TABLE S2, DOCX file, 0.02 MB. Copyright © 2018 Wydau-Dematteis et al. 2018 Wydau-Dematteis et al. This content is distributed under the terms of the Creative Commons Attribution 4.0 International license.

To check that the lytic activity detected as a 70-kDa band was associated with Cwp19, we generated a C. difficile *cwp19* mutant using a ClosTron system (see Fig. S1 in the supplemental material). Zymogram analysis of surface proteins of the *cwp19* mutant strain revealed the loss of the major 70-kDa lytic band present in the wild type. The minor band of 50 kDa was also absent in the mutant strain, suggesting that this band was due to an active Cwp19 degradation product (Fig. 1A). In contrast, the middle 55-kDa band was still present, indicating the presence of another active peptidoglycanase. Complementation of the *cwp19* mutant with a plasmid harboring the wild-type copy of the *cwp19* gene expressed with its promoter restored the major and the minor hydrolytic bands observed in the 630Δ*erm* strain (Fig. 1A), thus confirming that Cwp19 is responsible for the major lytic band observed on the zymogram. Therefore, our data demonstrate that Cwp19 is a peptidoglycanase, a result recently reported by Bradshaw et al. from preliminary activity tests (43).

10.1128/mBio.00648-18.1 FIG S1  Inactivation of Cwp19-encoding gene in C. difficile 630Δ*erm* using the ClosTron system. (A) Schematic presentation of pMTL-based knockout plasmid (a), wild-type *cwp19* gene (b), and mutated *cwp19* gene (c). The group II intron (black arrow), internal RAM conferring erythromycin resistance (white arrow), group I intron (light gray box), and *cwp19* gene (dark gray arrow) are represented. The ClosTron insertion site is shown with a vertical arrow. The locations of primers used for screening mutant and the size of expected PCR products are indicated. (B) Confirmation of gene knockouts using PCR with primers cwp19F and cwp19R. Download FIG S1, TIF file, 0.4 MB. Copyright © 2018 Wydau-Dematteis et al. 2018 Wydau-Dematteis et al. This content is distributed under the terms of the Creative Commons Attribution 4.0 International license.

### Cwp19 has lytic transglycosylase specificity.

To provide further evidence that Cwp19 functions as a PG-degrading enzyme and to examine its hydrolytic activity, we first attempted to produce and purify a recombinant His_6_-tagged Cwp19 protein in Escherichia coli. However, despite multiple attempts and diverse constructions, purification of recombinant Cwp19 resulted in poor yields and multiple degradation products. Similar Cwp19 purification issues in E. coli were previously reported (44). Therefore, we decided to overexpress Cwp19 in C. difficile using an inducible system. Cwp19 was expressed with its native signal peptide and a C terminus His_6_ tag on a plasmid under the control of the inducible *P*_*tet*_ promoter in C. difficile 630Δ*erm*. Recombinant His_6_-tagged Cwp19 was thus successfully purified and visualized as a single protein band near 70 kDa in SDS-PAGE. The identity of the purified His_6_-Cwp19 was further confirmed by Western blotting with anti-His_6_ antibodies (Fig. 1C). Moreover, purified His_6_-Cwp19 caused a strong zone of clearing in a zymogram assay using M. lysodeikticus cells as the substrate, confirming the PG-degrading activity of the purified Cwp19 protein (Fig. 1C).

**FIG 2:**
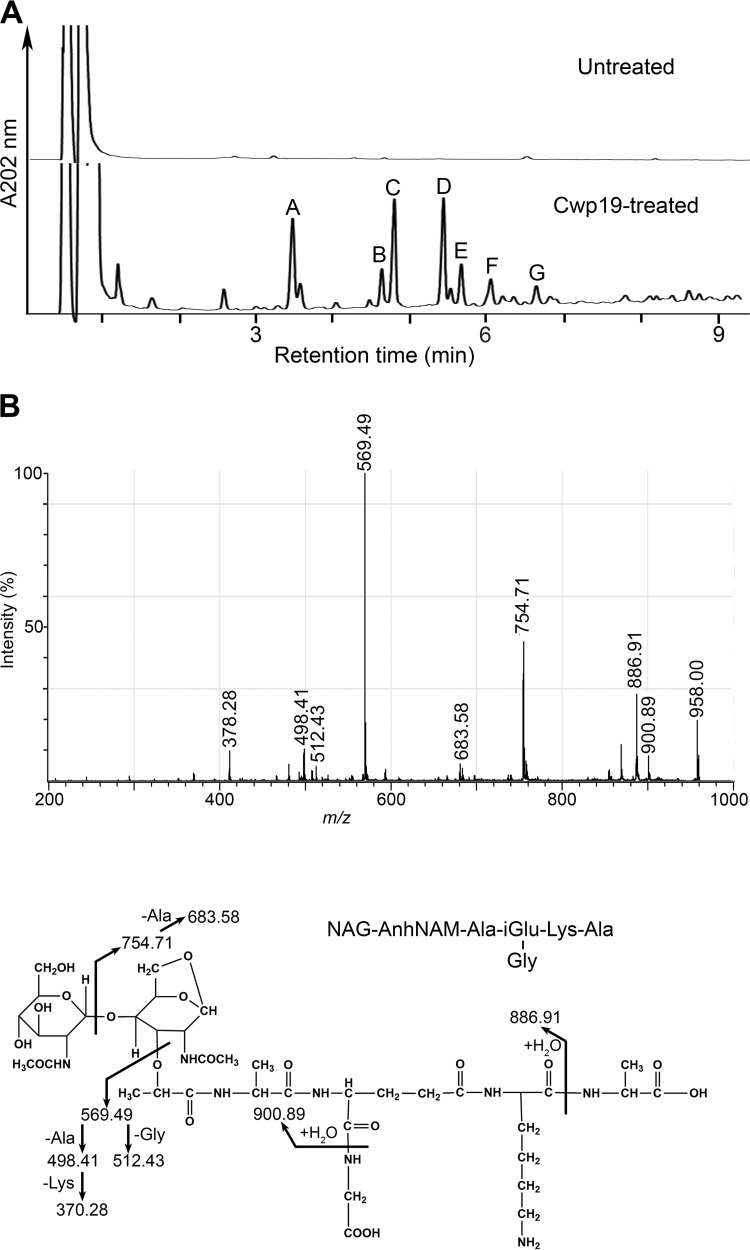
Cwp19 is a lytic transglycosylase. (A) RP-HPLC separation of soluble PG fragments released from M. lysodeikticus PG after incubation with buffer (Untreated) or His_6_-Cwp19 (Cwp19-treated). Annotated peaks were collected and their masses determined by MALDI-TOF MS. (B) MALDI-TOF/TOF tandem MS spectrum of the reaction product in peak C. The [M+Na]^+^ parent ion (*m*/*z* 957.55) was selected for fragmentation, and the inferred structure is represented at the bottom of the panel. The fragments were also detected as sodiated ions.

The cleavage specificity of Cwp19 was then determined by assaying the activity of the purified His_6_-Cwp19 on vegetative PG of C. difficile 630Δ*erm*. However, no peaks were observed when reaction supernatants were analyzed by reverse-phase high-pressure liquid chromatography (RP-HPLC), indicating that no soluble PG fragments had been generated. We reasoned that since His_6_-Cwp19 could lyse M. lysodeikticus cells in a zymogram assay, PG of M. lysodeikticus might constitute a suitable substrate to characterize the cleavage specificity of Cwp19. Therefore, purified PG of M. lysodeikticus was incubated with purified His_6_-Cwp19. RP-HPLC separation of the soluble fraction revealed the presence of several peaks (Fig. 2A). In contrast, no peaks were detected in a control reaction with M. lysodeikticus PG incubated without adding His_6_-Cwp19 (Fig. 2A). The main reaction products (peaks A to G in Fig. 2A) were analyzed by matrix-assisted laser desorption ionization–time of flight mass spectrometry (MALDI-TOF MS) and by MALDI-TOF/TOF tandem MS (MS/MS), and the resulting data were used to predict consistent structures (Table 1). Two muropeptides (present in peaks C and E) with *m*/*z* values of 957.55 and 1,028.59, respectively, were identified as 1,6-anhydromuropeptides bearing a terminal nonreducible 1,6-anhydroNAM residue. This proposed structure was deduced from the observation of appropriate product ions in the corresponding MS/MS spectrum (Fig. 2B; see also Fig. S2). The muropeptide in peak B had the same *m*/*z* value as another species also present in peak C and was therefore proposed to have the same structure. The occurrence of 1,6-anhydromuramoyl-containing muropeptides in the PG digestion products indicates that Cwp19 has LT activity. Interestingly, no other structure among the other characterized PG fragments was shown to contain a terminal 1,6-anhydroNAM residue. Regarding the products below peaks D and G, their *m*/*z* values were consistent with those of tetrasaccharidic structures with a reducing end and bearing a single peptidic side chain (Table 1). The MS/MS fragmentation patterns of these two parent ions were in line with the proposed structures and further indicated that the peptide chains of both muropeptides might be linked to the internal NAM of the tetrasaccharide chain (Fig. S3A and B). Both the amino acid composition of the stem peptides and the presence of unsubstituted NAM are in agreement with the structure of the cell wall PG of M. lysodeikticus established by Schleifer and Kandler (45). Regarding peaks A and F, MS/MS analysis indicated that the corresponding PG fragments might contain tetrasaccharides with an unsubstituted NAM reducing end (data not shown).

10.1128/mBio.00648-18.2 FIG S2  MALDI-TOF/TOF tandem MS spectrum of the reaction product in peak E. The [M+Na]^+^ parent ion (*m*/*z* 1,028.59) was selected for fragmentation, and the inferred structure is represented at the bottom of the panel. The fragments were also detected as sodiated ions. Download FIG S2, TIF file, 0.3 MB. Copyright © 2018 Wydau-Dematteis et al. 2018 Wydau-Dematteis et al. This content is distributed under the terms of the Creative Commons Attribution 4.0 International license.

10.1128/mBio.00648-18.3 FIG S3  MALDI-TOF/TOF tandem MS spectrum of the reaction product in peaks D (A) and G (B). (A) The [M+Na]^+^ parent ion (*m*/*z* 1,455.79) was selected for fragmentation, and the inferred structure is represented at the bottom of the panel. The fragments were also detected as sodiated ions. (B) The [M+Na]^+^ parent ion (*m*/*z* 1,583.93) was selected for fragmentation, and the inferred structure is represented at the bottom of the panel. The fragments were also detected as sodiated ions. Download FIG S3, TIF file, 0.9 MB. Copyright © 2018 Wydau-Dematteis et al. 2018 Wydau-Dematteis et al. This content is distributed under the terms of the Creative Commons Attribution 4.0 International license.

**TABLE 1:** Proposed structures and molecular masses of PG fragments resulting from the digestion of M. lysodeikticus PG by purified His_6_-Cwp19

Peak^a^	Proposed structure^b^	*m*/*z* ([M+Na]^+^)^c^	
		Observed	Calculated
B	NAG-AnhNAM-l-Ala-d-iGlu-(Gly)l-Lys-d-Ala	957.55	957.40
C	NAG-AnhNAM-l-Ala-d-iGlu-(Gly)l-Lys-d-Ala	957.55	957.40
D	NAG-NAM-(l-Ala-d-iGlu-[Gly]l-Lys-d-Ala)NAG-NAM	1,455.79	1,455.61
E	NAG-AnhNAM-l-Ala-d-iGlu-(Gly)l-Lys-d-Ala-(d-Ala)	1,028.59	1,028.44
G	NAG-NAM-(l-Ala-d-iGlu-[Gly]-l-Lys-[d-Ala-l-Lys])NAG-NAM	1,583.93	1,583.70

a Peak letters correspond to Fig. 2.

b The structures were deduced according to the previously determined structure of M. lysodeikticus PG (45). NAG, *N*-acetylglucosamine; NAM, *N*-acetylmuramic acid; AnhNAM, 1,6-anhydroNAM; iGlu, isoGlu.

c Sodiated molecular ions are the most abundant on MALDI-TOF mass spectra for all PG fragments. *m*/*z* values correspond to monoisotopic masses.

In summary, we identified the typical anhydromuropeptides resulting from LT activity in addition to several reaction products containing a reducing end (compounds corresponding to peaks A, D, F, and G). Similarly, PG fragments with a terminal reducing end have been previously reported to be reaction products of Bacillus subtilis and E. coli LTs, especially for enzymes exhibiting low activity *in vitro* (46–48). These products are proposed to be formed when the transient oxocarbenium intermediate entraps a water molecule instead of the C6-hydroxyl group of NAM, giving rise to hydrolytic cleavage of the NAM-NAG glycosidic bond (muramidase activity) rather than to nonhydrolytic cleavage resulting in anhydroNAM formation (LT activity). Therefore, we conclude that Cwp19 is an LT on the basis of our overall results. Interestingly, the reducing end-containing products that we identified as Cwp19 digestion products have a terminal unsubstituted NAM. We infer that the absence of peptide stem might interfere with the normal reaction mechanism of the LT and might lead to hydrolytic cleavage of the glycosidic bond.

### Transcription of *cwp19* during growth.

**FIG 3:**
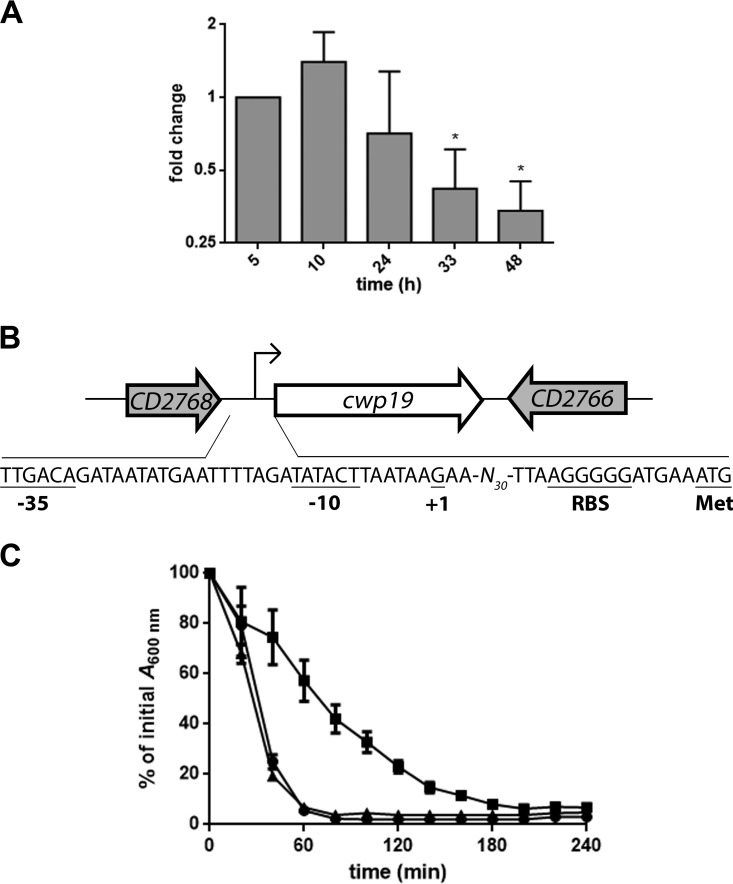
Expression and autolytic activity of Cwp19 in BHI medium. (A) qRT-PCR analysis of *cwp19* expression during cellular growth in BHI medium. Total RNAs were extracted from C. difficile 630Δ*erm* grown in BHI medium at different times of growth. cDNAs were prepared and qRT-PCR was performed using gene-specific primers as outlined in Materials and Methods. Cells incubated for 5 h served as the reference condition. *, *P* ≤ 0.05 (Student’s *t* test). (B) Schematic representation of the genetic organization of the *cwp19* chromosomal region of C. difficile strain 630Δ*erm*. Large arrows represent the genes, and their orientation shows the transcriptional direction. The nucleotide sequence of the *cwp19* promoter region is shown. The transcriptional initiation nucleotide (+1) identified by 5′ RACE, the putative ribosome binding site sequence, the −10 and −35 motifs, and the translational initiation codon are underlined. (C) Triton X-100-induced autolysis of the C. difficile wild type (●), *cwp19* mutant (■), and complemented *cwp19* mutant (▲) grown in BHI medium. The strains were harvested in the exponential-growth phase and transferred in 50 mM potassium phosphate buffer (pH 7.0) containing 0.01% Triton X-100. Autolysis was monitored by measuring the decrease of the bacterial suspension at OD_600_ and is expressed as a percentage of the initial OD_600_ value. Error bars indicate standard deviations. Values are given as means ± standard deviations (*n* = 3).

The *cwp19* mRNA levels were monitored during the bacterial growth in BHI medium by quantitative reverse transcription-PCR (qRT-PCR). Variation in *cwp19* expression was limited over time, with maximum expression at 10 h and the lowest level at 48 h (Fig. 3A). The *cwp19* promoter was mapped by rapid amplification of cDNA ends (RACE)-PCR. The apparent transcriptional start site of *cwp19* was located 47 bases upstream of its translational start codon (Fig. 3B). It was previously reported that the genes encoding the major autolysins LytC, LytD, and LytF in B. subtilis are transcribed by the RNA polymerase containing the alternative sigma factor σ^D^ (49, 50). However, we could not identify the described consensus sequence of C. difficile σ^D^-dependent promoters upstream of the transcriptional start site of *cwp19* (51). We found instead putative −10 (TATACT) and −35 sequences (TTGACA), resembling those of σ^A^-dependent promoters. In addition, expression of *cwp19* was not affected in a σ^D^ mutant (51). Overall, these results suggest that *cwp19* is primarily transcribed by σ^A^.

### Impact of *cwp19* inactivation on cell separation and autolysis.

Light microscopy analysis of cells in the exponential-growth phase revealed no effect on daughter cell separation in the absence of Cwp19 (data not shown). To determine if Cwp19 functions as an autolysin, a Triton X-100-induced-autolysis assay was performed on cells harvested during exponential growth in BHI medium. As shown in Fig. 3C, cellular lysis was rapid when the wild-type cells were resuspended in the presence of 0.01% Triton X-100. The autolysis of the *cwp19* mutant strain was slower than that of the wild-type strain, while complementation of the *cwp19* mutant restored the wild-type phenotype (Fig. 3C). Thus, Cwp19 appears to be a major autolysin of C. difficile.

### Cwp19 is involved in stationary-phase autolysis in BHI medium.

**FIG 4:**
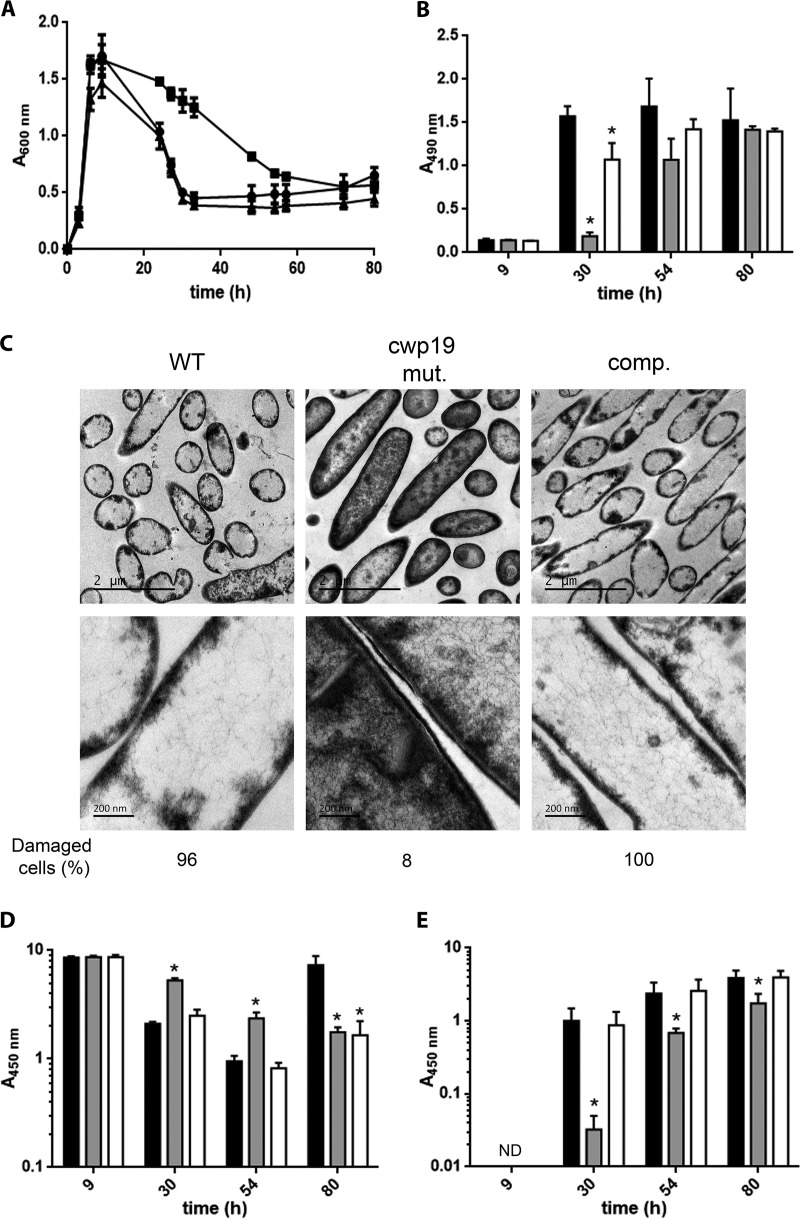
Role of Cwp19 in cell autolysis and toxin release of C. difficile cultivated in BHI medium. (A) Growth and autolysis of the wild-type 630Δ*erm* (●), *cwp19* mutant (■), and complemented *cwp19* mutant (▲) strains in BHI medium. Values are given as means ± standard deviations (*n* = 3). (B) LDH activity released in the supernatant fractions of wild-type 630Δ*erm* (black bars), *cwp19* mutant (gray bars), and complemented *cwp19* mutant (white bars) strains in BHI medium at different time points. LDH activity was determined using Promega CytoTox 96. The signal from the test was recorded as absorbance at 490 nm. Values are given as means ± standard deviations (*n* = 3). *, *P* ≤ 0.05 (Student’s *t* test). (C) TEM micrographs of C. difficile wild-type 630Δ*erm*, *cwp19* mutant (*cwp19* mut.), and complemented *cwp19* mutant (comp.) strains incubated for 33 h in BHI medium. The percentage of damaged cells for each strain is indicated. (D and E) Toxin titers in the cytosol (D) and in the culture supernatant (E) of wild-type 630Δ*erm* (black bars), *cwp19* mutant (gray bars), and complemented *cwp19* mutant (white bars) strains grown in BHI medium. Toxins were quantified by ELISA. The signal from the test was recorded as absorbance at 450 nm. Values are given as means ± standard deviations (*n* = 3). *, *P* ≤ 0.05 (Student’s *t* test). ND, not detectable.

C. difficile 630Δ*erm* grown in BHI medium lysed dramatically during the stationary phase as revealed by the fast decrease of the optical density at 600 nm (OD_600_) of the bacterial culture (Fig. 4A) (14). To test whether Cwp19 was involved in the observed stationary-phase autolysis, we monitored growth of the wild-type 630Δ*erm* strain and the *cwp19* mutant in BHI medium (Fig. 4A). The two strains exhibited the same growth rate and had similar behavior until 9 h after inoculation. At this point, the wild-type strain showed a rapid decrease of OD_600_ with a minimal OD_600_ reached after 33 h of incubation in BHI medium. A strong delay in cell lysis was observed in the *cwp19* mutant, which required 72 h of incubation to reach the same minimal OD_600_ as the wild-type strain. A similar decline phase was observed in comparisons between the complemented strain and the wild-type strain. We noted that the complemented strain had a growth defect compared to the wild type, reaching a lower final OD_600_. This was probably due to the higher level of Cwp19, expressed from a multicopy plasmid, causing premature cell lysis during the exponential growth. Similar growth and lysis features were obtained when the strains were grown for 100 h in an automatic plate reader (Fig. S4A).

10.1128/mBio.00648-18.4 FIG S4  Impact of growth medium on Cwp19-dependent autolysis. Growth of the C. difficile wild-type (blue lines), *cwp19* mutant (red lines), and complemented *cwp19* mutant (green lines) strains in BHI medium (A), TY medium (B), or TY medium supplemented with 0.2% glucose (C) at 37°C are shown. Growth curves were obtained using a GloMax plate reader (Promega). Values are given as means (*n* = 3). Download FIG S4, TIF file, 2.2 MB. Copyright © 2018 Wydau-Dematteis et al. 2018 Wydau-Dematteis et al. This content is distributed under the terms of the Creative Commons Attribution 4.0 International license.

To confirm that the OD decrease was a direct consequence of bacteriolysis, the release of cytosolic lactate dehydrogenase (LDH) into the supernatant was followed over time (Fig. 4B). In agreement with the autolysis profiles, the levels of extracellular LDH activity were lower in the *cwp19* mutant than in the wild type and the complemented strains at 30 and 54 h.

To explore the possible link between cell lysis and cell wall integrity, we analyzed by transmission electron microscopy (TEM) the wild-type, *cwp19* mutant, and complemented strains grown for 33 h in BHI broth (i.e., when the difference of OD_600_ was the biggest; see Fig. 4A). In the wild-type strain, almost all (96%) of the cells observed were lysed cells with an extremely thin cell wall and emptied of their cytoplasmic content (Fig. 4C). In contrast, the cell wall of the *cwp19* mutant cells was intact and well defined, and almost no (8%) lysed cells were observed. As expected, the complemented *cwp19* mutant showed the same thin cell walls and wall perforations as the wild-type strain (Fig. 4C). Taken together, these results demonstrate that Cwp19 plays a major role in stationary-phase autolysis of C. difficile cells when the cells are grown in BHI medium.

### Toxin release is mediated by Cwp19 autolysin in BHI medium.

It has previously been suggested that bacteriolysis is responsible for the release of toxins A and B of C. difficile 630Δ*erm* (14). Interestingly, all experiments in the study by Olling et al. were conducted in BHI medium. To determine the impact of Cwp19 on toxin release, the toxin levels in the cell cytosol and culture supernatants were monitored in the wild type and in the *cwp19* mutant and its complemented strain at different times of growth in BHI medium. For all strains, toxins were produced after 9 h of growth and their localization was intracellular (Fig. 4D and E). However, the location of the toxin for the wild-type strain shifted from the cytosol to the culture supernatant after 30 h of growth, which correlated with the cell lysis observed in Fig. 4A. In contrast, toxin release into the supernatant was strongly delayed in the *cwp19* mutant (Fig. 4D and E). The release pattern recorded for the wild type was also observed for the complemented *cwp19* mutant strain, which is in agreement with its autolysis profile (Fig. 4A, D, and E). Surprisingly, an increase in the intracellular levels of toxins was observed between 54 and 80 h for the wild-type strain (Fig. 4D). This secondary toxin production was associated with a slight increase in the OD_600_ (Fig. 4A) that might have been due to the growth of surviving cells using lysed cells, as cytoplasmic content can act as a source of nutrients in a nutrient-depleted environment.

Taken together, these results show that Cwp19 plays a critical role in toxin release of C. difficile 630Δ*erm* cultivated in BHI medium.

### Toxin release is independent of Cwp19-induced autolysis in TY medium.

**FIG 5:**
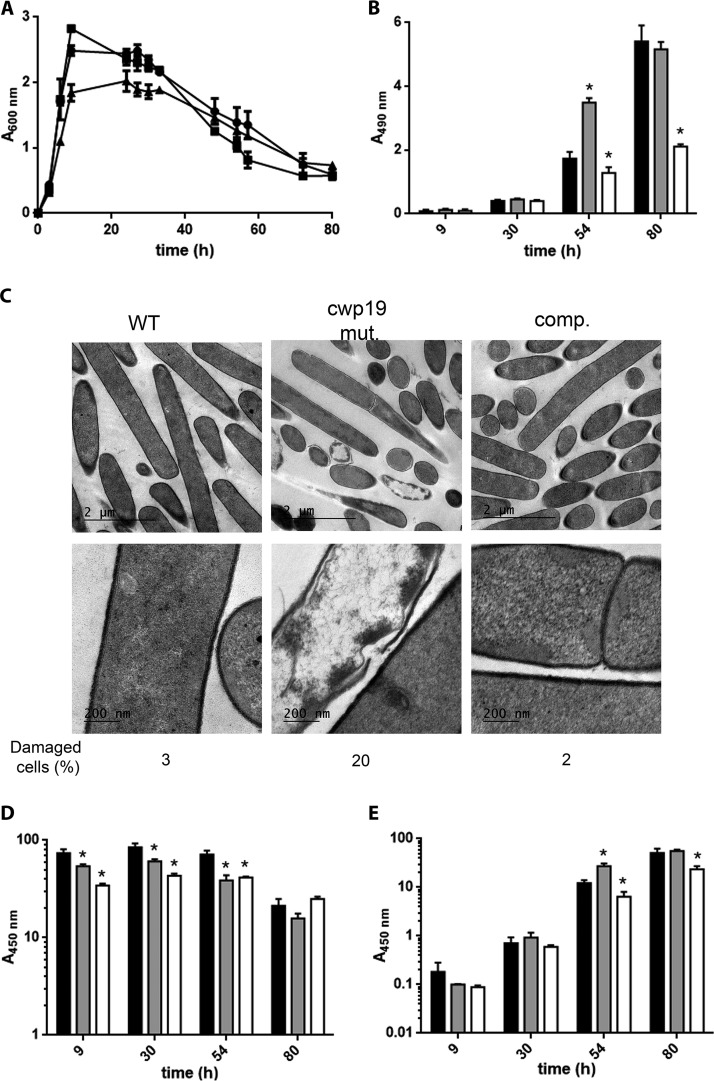
Role of Cwp19 in cell autolysis and toxin release of C. difficile cultivated in TY medium. (A) Growth and autolysis of wild-type 630Δ*erm* (●), *cwp19* mutant (■), and complemented *cwp19* mutant (▲) strains in TY medium. Values are given as means ± standard deviations (*n* = 3). (B) LDH activity released in the supernatant fractions of wild-type 630Δ*erm* (black bars), *cwp19* mutant (gray bars), and complemented *cwp19* mutant (white bars) strains in TY medium at different time points. LDH activity was determined using Promega CytoTox 96. The signal from the test was recorded as absorbance at 490 nm. Values are given as means ± standard deviations (*n* = 3). *, *P* ≤ 0.05 (Student’s *t* test). (C) TEM micrographs of C. difficile wild-type 630Δ*erm*, *cwp19* mutant (*cwp19* mut.), and complemented *cwp19* mutant (comp.) strains incubated for 48 h in TY medium. The percentage of damaged cells for each strain is indicated. (D and E) Toxin titers in the cytosol (D) and in the culture supernatant (E) of wild-type 630Δ*erm* (black bars), *cwp19* mutant (gray bars), and complemented *cwp19* mutant (white bars) strains grown in TY medium. Toxins were quantified by ELISA. The signal from the test was recorded as absorbance at 450 nm. Values are given as means ± standard deviations (*n* = 3). *, *P* ≤ 0.05 (Student’s *t* test).

In apparent discrepancy with the results presented in this study and those of Olling et al. (14), Govind and Dupuy have shown that secretion of C. difficile toxins A and B is mediated by the holin-like TcdE protein, independently of the bacterial cell lysis (12). However, it is worth noting that TY medium instead of BHI medium was used in the latter study. Thus, we sought to determine the impact of Cwp19 activity in toxin release from cells cultivated in TY medium. We observed that cell autolysis of the wild-type strain was much slower in TY medium than in BHI medium (Fig. 4A and 5A). The *cwp19* mutant did not show delayed autolysis compared to the wild type, indicating that the cell lysis occurring in TY medium was largely Cwp19 independent. Surprisingly, mutation of *cwp19* rather slightly accelerated cell lysis after 48 h of incubation (Fig. 5A). The same lysis kinetic as was seen with the wild type was observed in the complemented *cwp19* mutant strain, even though the maximal OD_600_ reached in this strain was lower. The same conclusions could be drawn when strains were grown in TY medium for 100 h in an automatic plate reader (Fig. S4B).

LDH levels in the supernatants of the three strains were in complete agreement with their lysis profiles and were slightly higher in the *cwp19* mutant than in the wild-type strain at 54 h (Fig. 5B). In addition, TEM was performed on cells cultivated in TY medium for 48 h, the time point when cell lysis occurs, which is different between the wild type and the mutant strain. While most of the cells of the wild-type and complemented strains were intact, a subpopulation of lysed cells was observed in the *cwp19* mutant (Fig. 5C). Thus, the absence of Cwp19 seems to stimulate the activity of another nonidentified PGH in TY medium.

Both intracellular toxin levels and accumulations of toxins in the supernatant were correlated with cell lysis (Fig. 5D and E). Most of the toxin release occurred after prolonged incubation times and could be attributed to Cwp19-independent cell lysis (Fig. 5A and E). However, low but significant toxin levels were detected in the extracellular medium after 9 h in TY medium. Cell lysis does not occur at that time, suggesting that early toxin release might be mediated by a TcdE-dependent secretion mechanism (12). The *cwp19* mutant released more toxins into the supernatant than the wild type after 54 h, in agreement with cell lysis results. Notably, the *cwp19* mutant strain also produced slightly lower total levels of toxins than the wild type after 9 h of incubation (Fig. 5D).

### Cwp19 is produced and active in cells grown in TY medium.

Since Cwp19 does not contribute to cell autolysis in TY medium, we sought to determine whether Cwp19 was produced and active under these conditions. Analysis by qRT-PCR revealed that *cwp19* is transcribed during the bacterial growth in TY medium (data not shown). Zymogram analysis of surface proteins of the wild-type and *cwp19* mutant strains grown in TY medium gave profiles of lytic bands (Fig. S5A) similar to the one observed in BHI medium (Fig. 1A). The 70-kDa and 50-kDa lytic bands corresponding to Cwp19 detected in the wild type disappeared in the *cwp19* mutant, confirming that Cwp19 is produced in an active form in TY medium. Furthermore, Triton X-100-induced autolysis of the wild-type, *cwp19* mutant, and complemented *cwp19* mutant strains was tested. Autolysis of the *cwp19* mutant strain was dramatically slower than that of the wild-type strain or the complemented *cwp19* strain (Fig. S5B). Thus, Cwp19 is synthesized and active when C. difficile 630Δ*erm* is cultivated in TY medium but does not play a major role in cell autolysis or, consequently, in toxin release during the stationary phase.

10.1128/mBio.00648-18.5 FIG S5  Activity of Cwp19 in TY medium. (A) Detection of bacteriolytic activities in surface protein extracts of the C. difficile 630 Δ*erm* (WT) and *cwp19* mutant (*cwp19* mut.) strains by zymogram. The gel contained 0.2% (wt/vol) M. lysodeikticus cells as a substrate. Surface proteins were extracted from cells grown to the exponential-growth phase in TY medium. Arrows indicate the positions of the bacteriolytic bands. (B) Triton X-100-induced autolysis of the C. difficile wild-type (●), *cwp19* mutant (■), and complemented *cwp19* mutant (▲) strains grown in TY medium. The strains were harvested in the exponential-growth phase and transferred in 50 mM potassium phosphate buffer (pH 7.0) containing 0.01% Triton X-100. Autolysis was monitored by measuring the OD_600_ decrease of the bacterial suspension and expressed as a percentage of the initial OD_600_ value. Error bars indicate standard deviations. Values are given as means ± standard deviations (*n* = 3). Download FIG S5, TIF file, 2.2 MB. Copyright © 2018 Wydau-Dematteis et al. 2018 Wydau-Dematteis et al. This content is distributed under the terms of the Creative Commons Attribution 4.0 International license.

### Glucose influences Cwp19-dependent lysis.

Both BHI and TY are rich media, but BHI medium contains 0.2% glucose. To examine whether the presence of glucose might impact Cwp19-mediated lysis, wild-type and *cwp19* mutant strains were grown for 100 h in TY medium supplemented with 0.2% glucose (TYG) and growth was monitored in an automatic plate reader (Fig. S4C). While Cwp19 did not induce cell autolysis in TY medium (Fig. S4B), the *cwp19* mutant lysed less than the wild-type strain in TYG. Therefore, this result indicates that glucose plays an important role in the control of Cwp19-dependent lysis, possibly by modifying cell surface features and thus facilitating access of Cwp19 to its PG substrate.

## DISCUSSION

In this study, we characterized a surface protein of C. difficile (Cwp19) with PG-degrading activity and we showed that it is involved in autolysis and toxin release after stationary-phase entry. Cwp19 has a novel catalytic domain named GHL10, previously known as DUF187 (41), which does not belong to any known domains specialized in cleavage of PG bonds in *Firmicutes* (see Table S1 in the supplemental material) (18). Thus, we report for the first time that the GHL10 domain, which is widely distributed in bacterial species, is a novel domain conferring PG-degrading activity and, more specifically, LT activity.

The first LT was discovered more than 40 years ago in E. coli (52), and six different catalytic domains with LT activity have since been identified in Gram-negative bacteria (53). In contrast, only a few LTs have been characterized in Gram-positive bacteria. Most of them are specific to spore-forming bacteria and are involved in the processes of spore formation or germination (53). In this study, we demonstrated that Cwp19 is synthesized in C. difficile vegetative cells and that Cwp19 activity leads to autolysis in stationary phase. Cwp19 does not have any sequence homology to known LTs and therefore represents the founding member of a widely distributed family of LTs.

Cwp19 belongs to the large family of C. difficile cell wall proteins (CWPs) comprising 29 members (41, 54). These proteins contain three tandem CWB2 motifs (Pfam04122), which mediate the attachment of CWPs to the cell wall through interactions with the anionic polymer PSII (55, 56). Several CWPs have been previously characterized in C. difficile. These include the S-layer proteins, the Cwp2 and Cwp66 adhesins, the phase-variable CwpV protein, and the Cwp13 and Cwp84 proteases (54, 57–59). Although the exact role of each CWP in pathogenesis remains to be elucidated, antibodies to many CWPs have been found in serum samples from C. difficile-infected patients, implying that certain CWPs are expressed and surface exposed *in vivo* (60, 61). Cwp19 is one of the CWPs present at the cell surface *in vivo* and expressed during the early step of colonization (61, 62). This suggests that Cwp19 could have an important role during the infection process of C. difficile.

While purified His_6_-Cwp19 readily digested M. lysodeikticus PG, it failed to generate a soluble fragment from PG purified from C. difficile. We believe this could have been due to structural modifications in the PG substrate, which are known to affect peptidoglycanase activity (63). Glycan chains of PG of C. difficile exhibit very high (approximately 93%) levels of NAG deacetylation (64). The degree of NAG deacetylation modulates lysozyme resistance in C. difficile (65), as well as in many Gram-positive bacteria (66). Moreover, NAG deacetylation has been shown to modulate activity of endogenous PGHs in bacteria, such as AcmA in Lactococcus lactis or LytA in S. pneumoniae (67, 68). All PG fragments generated from digestion of M. lysodeikticus PG contained acetylated NAG residues, and it is therefore possible that Cwp19 would not cleave the β(1-4) bond between a NAM and an *N*-unsubstituted glucosamine. According to this model, C. difficile PG, which contains only low levels of NAM-NAG glycosidic bonds, would be cleaved to a much lesser extent by Cwp19. Thus, soluble PG fragments are probably generated but such fragments are likely too large for HPLC analysis. How could Cwp19 activity lead to cell autolysis while having such limited cleavage sites in C. difficile PG? One hypothesis is that the rare NAG residues might not be randomly distributed around the cells but rather might be randomly distributed at key locations and that the cleavage of the NAG-NAM glycosidic bonds would destabilize the entire PG network. An alternative possibility is that Cwp19 makes small breaches inside the PG network, facilitating access for other PG-degrading enzymes.

In S. pneumoniae, the major autolysin LytA, with *N*-acetylmuramoyl-l-alanine amidase activity, causes dramatic autolysis of bacteria at the stationary phase (26, 69) and is thought to contribute to pneumococcal virulence by releasing the cytoplasmic content, including the pneumolysin toxin (26, 27). Interestingly, LytA does not induce cell lysis during the logarithmic-growth phase, although it is present at the cell surface, suggesting that the PG is somehow protected from the lytic activities of LytA (70). In our study, we have shown that Cwp19 is transcribed during the exponential-growth phase. Moreover, Triton X-100-induced autolysis from cells in the exponential phase of growth revealed an important and immediate role of Cwp19. Since Cwp19 is cell wall associated, these results suggest that Cwp19 cannot exert its influence on the PG during exponential growth, as observed for LytA in S. pneumoniae. The mechanism that initiates Cwp19-mediated cell wall degradation remains unclear but it is likely linked to cell wall modifications that occur when cells enter the stationary phase. Secondary cell wall polymers, including teichoic acids (wall teichoic acids and lipoteichoic acids) and polysaccharides, can modulate autolytic activity by shielding PG (71). In C. difficile 630, two types of surface polysaccharides have been described: the wall teichoic-like PSII acids and the PSIII lipoteichoic acid (72, 73). Thus, it is possible that protection of PG against Cwp19 might be conferred by one of these two polymers.

In BHI medium, Cwp19 is the main peptidoglycanase responsible for the C. difficile cell autolysis triggered at the beginning of the stationary phase. However, autolysis of the strain lacking Cwp19 was not completely abolished and lysis levels similar to those seen with the wild type were observed at later time points (Fig. 4A). Cwp19-independent lysis was also observed when bacteria were grown in TY medium (Fig. 5A). These observations indicate that Cwp19 is not the only autolysin involved in the lysis of C. difficile during the stationary phase and highlight the involvement of other PG-degrading enzymes. Functional redundancy of multiple autolysins has been demonstrated in B. subtilis (20, 74) and is consistent with the large number of putative autolysins present in C. difficile (Table S1). Interestingly, a lytic band was still present on the zymogram for the *cwp19* mutant and the corresponding unidentified enzyme is the preferential candidate for this persistent lysis. This enzyme is unlikely to be Acd, the first autolysin described in C. difficile (33), since inactivation of Acd had no impact on the bacteriolytic activity profile in a zymogram assay, on Triton X-100-induced autolysis, or on the stationary-phase autolysis in BHI medium (unpublished data).

The role of the holin-like TcdE protein in the release of C. difficile TcdA and TcdB has been controversial. On the one hand, it was previously shown that TcdE mediates toxin release in strain JIR8094 (also referred to as 630E) (12), an erythromycin-sensitive derivative of strain 630 (ribotype 012) (75) as well as in strain R20291 (ribotype 027) grown in TY medium (13). On the other hand, a *tcdE* inactivation did not affect toxin release in strain 630Δ*erm*, another erythromycin-sensitive derivative of strain 630 that has been shown to behave differently from strain JIR8094 (76) cultivated in BHI medium or from strain CD646 (ribotype 078) cultivated in TY medium. This result is mainly supported by the extensive autolysis that occurs during entry into the stationary phase in both strains (13, 14). The new model emerging from our findings is that Cwp19-dependent autolysis is the major mechanism responsible for toxin release *in vitro* when 630Δ*erm* cells are cultivated in BHI medium (Fig. 4) but not in TY medium (Fig. 5). Interestingly, when strain 630Δ*erm* was grown in TY medium, significant extracellular toxin levels were detected as early as 9 h, a time point where cell lysis was not occurring (Fig. 5E). This result reinforces the idea of a role of TcdE in toxin secretion under this growth condition (12). Notably, toxin production was approximately 9-fold higher when strain 630Δ*erm* was grown in TY medium than when it was grown in BHI medium (Fig. 4C and 5C). This result is in agreement with the repression of toxin expression by glucose through the carbon catabolic repression system (77, 78) and shows an inverse correlation between the abundance of toxins produced and the importance of Cwp19-mediated lysis for toxin release. TcdE-dependent secretion of toxin, which presents the benefit of preserving the cells from death, might be the preferred mechanism used by C. difficile when cells are in an environment supporting abundant toxin production. In contrast, the toxins released by TcdE in an environment inducing low toxin production would be insufficient and Cwp19-dependent release of toxins, an efficient but lethal mechanism, would then be required. In line with this hypothesis, we found that Cwp19-dependent lysis is also controlled by the presence of glucose (see Fig. S4C in the supplemental material).

In B. subtilis, autolytic amidases LytC, CwlC, and CwlH are required for hydrolysis of the mother cell wall peptidoglycan to release the mature endospore (79, 80). *In* C. difficile, Cwp19 cleaves the cell wall peptidoglycan after entry into the stationary phase and might thus play a role in sporulation or in mother cell lysis to release spores. However, even though inactivation of *cwp19* had a slight effect on sporulation efficiency (4% for the wild type and 1% for the mutant strain in BHI medium after 48 h of growth), the overall sporulation frequency of the 630Δ*erm* strain was low and free spores were observed in both the wild type and the *cwp19* mutant strain (data not shown). Additionally, most of the wild-type cells grown in BHI medium for 33 h were severely damaged as observed by TEM (Fig. 4C) but no spores could be observed, highlighting the lack of correlation between cell lysis and sporulation. Furthermore, *cwp19* is predicted to be transcribed by σ^A^ and does not depend on the σ^F^, σ^E^, σ^G^, and σ^K^ sporulation sigma factors (81). Hence, these data suggest that release of the endospores from the mother cells is not the primary purpose of Cwp19-mediated cell lysis.

What is the relevance of Cwp19 in toxin release when C. difficile colonizes the gut? Answering this question will require the deciphering of the mechanism controlling Cwp19 activity in response to the gut nutritional status as suggested by the differences observed *in vitro*. At this stage, we can assume only that Cwp19 and TcdE are coexisting mechanisms for toxin release acting independently in response to the nutritional changes occurring during the infection process. During the colonization phase, C. difficile does not need to compete for access to nutrients and the cells are probably in a state of continuous active growth. Therefore, cell lysis would not occur under these conditions and the release of toxins might be handled by the TcdE-dependent mechanism. As infection develops, nutrient availability becomes limited and such limitations would change the environmental conditions. These changes might induce Cwp19-mediated autolysis, leading to an important release of toxins. For this mechanism to have any benefit for C. difficile, it would be required to be asynchronous, with only a subpopulation of cells initiating it. Thus, the surviving population would profit from the release of nutrients due to the rupture of the intestinal epithelial cells. Severe diarrhea would be also induced, and this would contribute to the dissemination of the bacteria to new and potentially more supportive environments.

This study was focused solely on the 630Δ*erm* strain, but Cwp19 is present in all C. difficile strains that have been sequenced so far. We assume that this protein plays a conserved and important role in autolysis and toxin release in a wide range of strains. Cwp19 may represent an attractive drug target to prevent toxin release during CDI, and, as such, it would be highly relevant to expand the analysis of Cwp19 function to other clinically significant strains of C. difficile such as the R20291 strain. Finally, we demonstrated that Cwp19 PG-degrading activity is controlled by environmental conditions such as nutritional signals. Deciphering both the mechanism controlling the activity of Cwp19 and the nature of the signals also represents a promising avenue for another therapeutic approach targeting the induction of C. difficile cell autolysis before toxin production.

## MATERIALS AND METHODS

### Bacterial strains and culture conditions.

Bacterial strains and plasmids used in this study are listed in Table S3 in the supplemental material. C. difficile 630Δ*erm* (82) was used in all experiments. C. difficile strains were routinely cultured on BHI agar (Difco), BHI broth (Difco), or TY broth (77) at 37°C in an anaerobic environment (80% [vol/vol] N_2_, 10% [vol/vol] CO_2_, and 10% [vol/vol] H_2_). When necessary, C. difficile culture media were supplemented with cefoxitin (Cfx; 25 mg/liter), cycloserine (Ccs; 250 mg/liter), thiamphenicol (Tm; 7.5 mg/liter), and erythromycin (Erm; 5 mg/liter). E. coli strains were cultured at 37°C in LB broth or LB agar (MP Biomedicals), containing chloramphenicol (25 mg/liter) or ampicillin (100 mg/liter) when necessary.

10.1128/mBio.00648-18.8 TABLE S3  Strains, plasmids, and oligonucleotides used in this study. Download TABLE S3, DOCX file, 0.02 MB. Copyright © 2018 Wydau-Dematteis et al. 2018 Wydau-Dematteis et al. This content is distributed under the terms of the Creative Commons Attribution 4.0 International license.

### RNA isolation and quantitative real-time PCR.

C. difficile cultures were inoculated from overnight grown cells in TY or BHI medium at an OD_600_ of 0.05. Samples of total RNA were isolated from C. difficile cells with a FastRNA Pro Blue kit (MP Biomedicals). cDNA synthesis and real-time quantitative PCR were performed as previously described (83, 84). In each sample, the quantity of cDNAs of a gene was normalized to the quantity of cDNAs of the *dnaF* gene (*CD1305*) encoding DNA polymerase III. The relative change in gene expression was recorded as the ratio of normalized target concentrations (threshold cycle [ΔΔ*C*_*T*_] method) (85).

### Mapping of the transcriptional start site.

The 5′ end of *cwp19* mRNA was mapped from a 5′ rapid amplification of cDNA ends (RACE)-PCR product obtained with a 3′/5′ RACE kit (Roche Diagnostics). Briefly, the first-strand cDNA was synthesized from total RNA using primer sp1 (Table S3), avian myeloblastosis virus (AMV) reverse transcriptase, and the deoxynucleoside triphosphate (dNTP) mixture of the 3′/5′ RACE kit as recommended by the manufacturer. After purification and dG tailing of the cDNA, a nested PCR using the oligo(dC) anchor primer and the sp2 specific primer was carried out. The PCR product was purified and sequenced using primer sp3 (Table S3).

### Expression and purification of the Cwp19-His_6_-tagged fusion protein in C. difficile 630 Δ*erm*.

Primers cwp19surF/cwp19surR flanking *cwp19* (Table S3) were used for PCR amplification of the *cwp19* coding sequence and its preceding ribosome binding site (RBS) and to add a C-terminal His_6_ tag. Purified PCR product was digested with BamHI and XhoI and ligated into pRPF185, under the control of the anhydrotetracycline (ATc)-inducible *P*_*tet*_ promoter (86), and digested with the same enzymes, yielding pSUR19. Plasmid pSUR19 was initially transformed into E. coli Top10, and the sequence of the insertion was verified by sequencing. Plasmid was then transformed into the conjugative donor E. coli HB101 (RP4) and transferred via conjugation into C. difficile 630Δ*erm*. Cwp19 expression was induced in the presence of 500 ng/ml ATc for 4 h, and cells were then harvested by centrifugation. After cell disruption using a single freeze-thaw cycle as previously described (86), the soluble fraction containing the recombinant protein was purified by affinity chromatography with Talon metal affinity resin (Clontech). The pure protein was dialyzed and concentrated by ultrafiltration with Amicon Ultra-4 centrifugal filter units (Millipore) with a 30-kDa cutoff.

### Construction of C. difficile *cwp19* mutant and *cwp19* complemented strains.

To generate a *cwp19* mutant, a ClosTron system was used as described previously (87). Briefly, the algorithm available on the TargeTron design site was used to identify intron insertion sites within *cwp19*. The primers chosen to retarget the group II intron on pMTL007 to *cwp19* were used with EBS universal primer and intron template DNA to generate a 353-bp DNA fragment by overlap PCR (Table S3). The PCR product was cloned into the HindIII and BsrGI restriction sites of pMTL007. The sequence of the insertion was verified by sequencing using pMTL007-specific primers pMTLseq-F and pMTLseq-R (Table S3). Plasmid pMTL007::*cwp19*-588s retargeted the group II intron for insertion into the *cwp19* gene in the sense orientation immediately after nucleotide 588 in the coding sequence, i.e., the DNA sequence encoding the catalytic domain. This derivative pMTL007 plasmid was transformed into the conjugative donor E. coli HB101 (RP4) and then transferred via conjugation into the C. difficile 630Δ*erm* strain. Transconjugants were selected by subculturing on BHI agar containing Cfx, Ccs, and Tm and then plated on BHI agar containing Erm. Erm-resistant (and Tm-sensitive) C. difficile colonies were produced, following plasmid loss and insertion of the group II intron into the chromosome, which was accompanied by splicing out of the group I intron from the *ermB* retrotransposition-activated marker (RAM). Moreover, genomic DNA of Erm-resistant transconjugants was extracted and was subjected to PCR using primers flanking *cwp19* (cwp19 F and cwp19 R) (Table S3) to verify that the group II intron had inserted into the correct target gene. To complement the *cwp19* mutant, the *cwp19* gene with its own promoter was amplified by PCR using primers cwp19 compF and cwp19 compR (Table S3). The PCR fragment digested by XhoI and PvuI was cloned into pMTL007 previously digested with the same enzymes, and the resulting plasmid, pCOMP19, was introduced by conjugation into the C. difficile *cwp19* mutant strain, using E. coli HB101 (RP4) as a donor.

### Cell lysis and protein analysis.

Whole-cell lysates were prepared as previously described (86). For cell wall protein extraction, cultures of C. difficile were harvested by centrifugation at 5,000 × *g* for 10 min at 4°C, washed in phosphate buffer saline (PBS), and resuspended in SDS-PAGE sample buffer (88) to reach an OD_600_ of 20 and boiled for 10 min. For analysis by SDS-PAGE, an equal volume of 2× SDS sample buffer was added to protein samples (88). SDS-PAGE and Western immunoblotting were carried out using standard methods. Lytic activities were detected by zymography using SDS-polyacrylamide gels containing 0.2% (wt/vol) M. lysodeikticus ATCC 4698 cells (Sigma). After electrophoresis, the gel was shaken at 37°C for 16 h in 50 ml of 25 mM Tris-HCl (pH 8.0) solution containing 1% (vol/vol) Triton X-100 to allow protein renaturation. Clear bands resulting from lytic activity were visualized after staining with 1% (wt/vol) methylene blue (Sigma)–0.01% (wt/vol) KOH and subsequent destaining with distilled water.

### Protein identification by electrospray ionization (ESI)–LC-MS/MS.

The protein band of interest was excised from SDS-PAGE gels. The sample was reduced for 1 h at 50°C using 10 mM dithiothreitol (DTT) (GE Healthcare) and alkylated with 55 mM iodoacetamide (Sigma) for 1 h in the dark. Fragments were washed several times with water and ammonium carbonate, dehydrated with acetonitrile (ACN), and dried. Trypsin digestion was performed overnight with a dedicated automated system (MultiPROBE II; PerkinElmer). To allow extraction of peptides, the gel fragments were subsequently incubated twice for 15 min in a H_2_O/CH_3_CN solution, once for 15 min in 1% (vol/vol) formic acid, and once with 100% ACN. Supernatants were pooled into a clean 96-well plate. Peptide extracts were then dried and solubilized in starting buffer (3% CH_3_CN–0.1% formic acid–water) for chromatographic elution.

Peptides were analyzed using a nano-LC1200 system coupled to a 6340 Ion Trap mass spectrometer equipped with an HPLC-chip cube interface (Agilent Technologies, Massy, France). The tandem mass spectrometry peak lists were extracted using the DataAnalysis program (version 3.4, Bruker Daltonics) and compared with the protein database using the Mascot Daemon (version 2.1.3) search engine. The searches were performed with a maximum of one missed cleavage, with no fixed modifications, and with variable modifications for carbamidomethyl and oxidation of methionines. The identifications from the tandem mass spectrometry spectra were performed with mass tolerances of 1.6 Da for precursor ions and 0.8 for MS/MS fragments. The determination of at least two peptide sequences with a Mascot score of over 60 allowed a satisfactory identification of the protein.

### Toxin and lactate dehydrogenase (LDH) assays.

Total TcdA amounts were quantified in supernatants and cytosols from BHI and TY cultures by enzyme-linked immunosorbent assay (ELISA). Briefly, 1.5 ml of culture was taken and pelleted at the indicated time points by centrifugation for 4 min at 13,000 rpm. Bacterial pellets were frozen at −20°C. The supernatants were collected and subjected to ELISA. The frozen bacteria were thawed, resuspended in PBS, and incubated for 1 h at 37°C under conditions of agitation. Following sonication, the lysates were centrifuged for 3 min at 13,000 rpm to eliminate cell debris. Then, the intracellular toxins were subjected to ELISA. A 96-well immuno-plate (Nunc Maxisorp) was coated overnight at 4°C with 2 µg/ml of anti-toxin A rabbit polyclonal antibody (Abcam, Inc.). The coated wells were washed and incubated with Superblock blocking buffer (Thermo Fisher Scientific) for 1 h. The wells were then washed and air dried. Appropriate dilutions of samples were added into the wells, and the plate was incubated at 37°C for 90 min. After washings, 0.2 µg/ml of an anti-toxin A chicken horseradish peroxidase (HRP) antibody (LSBio) was added in each well and the plate was incubated for 1 h at 37°C. The wells were washed and incubated with a TMB (3,3',5,5'-tetramethylbenzidine) substrate solution (Thermo Fisher Scientific) for 15 min in the dark. A volume of stop solution (H_2_SO_4_; 0.2 M) was added into each well, and the signal from the test was recorded as absorbance at 450 nm.

LDH activity was determined using a CytoTox 96 kit from Promega according to the recommendations of the supplier.

### Autolysis assays.

For Triton X-100-induced autolysis, overnight cultures were diluted to an OD_600_ of 0.05 in BHI broth and grown at 37°C until the OD_600_ reached 0.5. Cells were harvested, washed twice, and suspended to an OD_600_ of 0.5 in 50 mM potassium phosphate buffer (pH 7.0) containing 0.01% Triton X-100. The OD_600_ of the bacterial suspension incubated at 37°C was then measured every 20 min to follow cell lysis.

### Determination of hydrolytic bond specificity.

PG from C. difficile 630Δ*erm* and M. lysodeikticus ATCC 4698 vegetative cells was prepared as described previously (64). Purified PG (1 mg dry weight) was digested with purified recombinant His_6_-Cwp19 protein (25 µg) in sodium phosphate buffer (50 mM; pH 6.0) for 24 h at 37°C with shaking. Samples were boiled for 3 min to stop the reaction, and the insoluble material was removed by centrifugation at 14,000 × *g* for 15 min. The soluble muropeptides obtained after digestion were reduced with sodium borohydride and were then separated by reverse-phase ultra-high-pressure liquid chromatography (RP-UHPLC) with a 1290 chromatography system (Agilent Technologies) and a Zorbax Eclipse Plus C_18_ RRHD column (Agilent Technologies) (100 by 2.1 mm; particle size, 1.8 µm) at 50°C using ammonium phosphate buffer and a methanol linear gradient (89). One microliter of collected muropeptides was then spotted directly on the MALDI target and thoroughly mixed with 1 µl of α-cyano-4-hydroxycinnaminic acid solution (5 mg/ml–50% ACN–0.1% trifluoroacetic acid [TFA]). Muropeptides were analyzed by MALDI-TOF MS with an UltrafleXtreme instrument (Bruker Daltonics, Bremen, Germany). MS spectra were acquired at a 2-kHz laser repetition rate in the positive reflector ion mode, with a 20-kV acceleration voltage and an extraction delay of 130 ns. The spectra are obtained by accumulating 1,000 to 5,000 shots (depending on the samples) over the 500 to 5,000 m/z range. Putative muropeptide structures were proposed based on MS/MS spectra acquired in LIFT mode at a 1-kHz laser repetition rate, applying 7.5 kV for initial acceleration of ions and 19 kV for reacceleration of fragments in the LIFT cell.

### Transmission electron microscopy.

Cells of C. difficile were collected after 33 h of growth in BHI medium or 24 h of growth in TY medium, centrifuged, and washed in PB {0.05 M PIPES [piperazine-N,N′-bis(2-ethanesulfonic acid)] buffer, 5 mM CaCl_2_, pH 7.3}. Each sample was fixed overnight at 4°C in PB containing 2.5% glutaraldehyde and 2% paraformaldehyde. The samples were postfixed first in PB–1% osmium tetroxide (45 min at 4°C) and then in 1% aqueous uranyl acetate solution for 2 h at room temperature. The samples were then dehydrated in increasing concentrations of ethanol (30%, 50%, 70%, 95%, and 100%) followed by ethanol/propylene oxide (1/1 [vol/vol]) and propylene oxide and were finally embedded in Epon epoxy resin. Ultrathin (80-nm) sections were stained with lead citrate and imaged at 80 kV in a Jeol-100S transmission electron microscope.
