# Supplementary material for: Cwp19 Is a Novel Lytic Transglycosylase Involved in Stationary-Phase Autolysis Resulting in Toxin Release in Clostridium difficile
Source: mBio. 2018 Jun 12;9(3):e00648-18. doi: 10.1128/mBio.00648-18 (PMC6016235; doi:10.1128/mBio.00648-18)
Supplement: TABLE S1 [file mbo003183933st1.docx]

**Table S1.** List of predicted PG-degrading enzymes identified in *C. difficile* 630.

| Protein family (PFAM)^1^ | | gene (name) | Signal peptide | | Molecular weight(kDa)^2^ | Cell-wall  binding domain^3^ | Features |
| --- | --- | --- | --- | --- | --- | --- | --- |
|  | **Endo- *N*-acetylglucosaminidase** | | | | | | |
| Glucosaminidase  (PF01832) | | *CD1034* | Yes | 48.4 | | - |  |
|  |  | *CD1304* (*acd*) | Yes | 63.6 | | SH3_3 X4 | Catalytic activity demonstrated ([1](#_ENREF_1)) |
|  |  | *CD2187* | No | 34.4 | | - | Putative phage-related enzyme |
|  |  | *CD2193* (*cwp24*) | Yes | 48.5 | | CWB2 X3 |  |
|  | ***N*-acetylmuramoyl-L-alanine amidase** | | | | | | |
| Amidase_3 (PF01520) | | *CD0106* | No | 26.9 | | - | Putative germination specific enzyme |
|  |  | *CD0784* | Yes | 29.6 | | - | SigG-dependent ([2](#_ENREF_2)) |
|  |  | *CD0972* | No | 30.1 | | - | Putative phage-related enzyme |
|  |  | *CD1035* (*cwp16*) | Yes | 71.1 | | CWB2 X3 |  |
|  |  | *CD1036* (*cwp17*) | Yes | 70.7 | | CWB2 X3 |  |
|  |  | *CD1898* | No | 28.6 | | - | Putative phage-related enzyme. SigK-dependent ([2](#_ENREF_2)) |
|  |  | *CD2761* | No | 33.9 | | - | SigE-dependent ([2](#_ENREF_2)) |
|  |  | *CD2784* (*cwp6*) | Yes | 70.4 | | CWB2 X3 |  |
|  |  | *CD2894* | No | 30.1 | | - | Putative phage-related enzyme |
| Hydrolase_2  (PF07486) | | *CD3563* (*sleB*) | Yes | 16.0 | | - | Putative spore-cortex lytic enzyme |
|  | **Endopeptidase** | | | | | | |
| NlpC/P60 (PF00877) | | *CD0183* | Yes | 34.5 | | SH3_3 X2 |  |
|  |  | *CD0372* | Yes | 33.4 | | - | Tn916-like,CTn1-Orf17 |
|  |  | *CD0961* | No | 74.3 | | - | Putative phage-related enzyme |
|  |  | *CD1135* | Yes | 42.3 | | SH3_3 X3 |  |
|  |  | *CD1368* | No | 57.0 | | - | Putative phage-related enzyme |
|  |  | *CD1857* | No | 83.0 | | - | Tn1549-like,CTn5-Orf13 |
|  |  | *CD2402* | Yes | 39.0 | | SH3_3 X3 | Putative phosphatase-associated protein |
|  |  | *CD2768* | Yes | 21.3 | | SH3_3 X1 |  |
|  |  | *CD2903* | No | 74.3 | | - | Putative phage-related enzyme |
|  |  | *CD3336* | No | 36.8 | | - | Tn916-like,CTn6-Orf13 |
|  |  | *CD3380* | Yes | 33.6 | | - | Tn916-like,CTn7-Orf16 |
| Peptidase_M23 (PF01551) | | *CD0125*  (*spoIIQ*) | No | 24.7 | | - | Characterized protein ([3](#_ENREF_3), [4](#_ENREF_4)) |
| CHAP^4^  (PF05257) | | *CD1108* | No | 71.7 | | - | Tn1549-like, CTn4-Orf11 |
|  | Lytic transglycosylase | | | | | | |
| SLT (PF01464) | | *CD1130* | No | 24.4 | | - |  |
|  |  | *CD0226* | No | 24.6 | | - |  |
| Hydrolase_2  (PF07486) | | *CD3563*  (*sleB*) | Yes | 15.8 | | - | Characterized protein ([5](#_ENREF_5)) |
| SpoIID  (PF08486) | | *CD0124*  (*spoIID*) | Yes | 36.4 | | - | Crystal structure  ([6](#_ENREF_6)) |
| No characteristic PFAM | | *CD0551* (*sleC*) | No | 47.3 | | PG_binding_1 X1 | Spore-cortex lytic enzyme, catalytic activity demonstrated ([7](#_ENREF_7)) |

^1^ PFAM database (http://www.sanger.ac.uk/resources/databases/pfam.html) was used to classify protein families. Carboxypeptidases are members of PGHs that do not induce bacteriolysis. As such, they are not listed in this table.

^2^Predicted signal peptides were excluded for the calculation of the theorical molecular weights.

^3^Cell wall binding domains SH3_3, CWBD2 and PG_binding_1 were assigned from PFAM accession numbers PF08239, PF04122 and PF01471. Symbol – indicates the absence of cell wall binding domain.

^4^ CHAP domain can function either as endopeptidase or amidase.

**REFERENCES**

1. **Dhalluin A, Bourgeois I, Pestel-Caron M, Camiade E, Raux G, Courtin P, Chapot-Chartier MP, Pons JL.** 2005. Acd, a peptidoglycan hydrolase of *Clostridium difficile* with *N*-acetylglucosaminidase activity. Microbiology **151:**2343-2351.

2. **Saujet L, Pereira FC, Serrano M, Soutourina O, Monot M, Shelyakin PV, Gelfand MS, Dupuy B, Henriques AO, Martin-Verstraete I.** 2013. Genome-wide analysis of cell type-specific gene transcription during spore formation in *Clostridium difficile*. PLoS Genet **9:**e1003756.

3. **Serrano M, Crawshaw AD, Dembek M, Monteiro JM, Pereira FC, de Pinho MG, Fairweather NF, Salgado PS, Henriques AO.** 2015. The SpoIIQ-SpoIIIAH complex of *Clostridium difficile* controls forespore engulfment and late stages of gene expression and spore morphogenesis. Mol Microbiol doi:10.1111/mmi.13311.

4. **Fimlaid KA, Jensen O, Donnelly ML, Siegrist MS, Shen A.** 2015. Regulation of *Clostridium difficile* Spore Formation by the SpoIIQ and SpoIIIA Proteins. PLoS Genet **11:**e1005562.

5. **Burns DA, Heap JT, Minton NP.** 2010. SleC is essential for germination of *Clostridium difficile* spores in nutrient-rich medium supplemented with the bile salt taurocholate. J Bacteriol **192:**657-664.

6. **Nocadello S, Minasov G, Shuvalova LS, Dubrovska I, Sabini E, Anderson WF.** 2016. Crystal Structures of the SpoIID Lytic Transglycosylases Essential for Bacterial Sporulation. J Biol Chem **291:**14915-14926.

7. **Gutelius D, Hokeness K, Logan SM, Reid CW.** 2014. Functional analysis of SleC from *Clostridium difficile*: an essential lytic transglycosylase involved in spore germination. Microbiology **160:**209-216.
