# Supplementary material for: Cwp19 Is a Novel Lytic Transglycosylase Involved in Stationary-Phase Autolysis Resulting in Toxin Release in Clostridium difficile
Source: mBio. 2018 Jun 12;9(3):e00648-18. doi: 10.1128/mBio.00648-18 (PMC6016235; doi:10.1128/mBio.00648-18)
Supplement: TABLE S2 [file mbo003183933st2.docx]

**Table S2.** Phylogenetic distribution of GHL10 domain.

| **BACTERIA** | | |
| --- | --- | --- |
| **Phylum Class** | **number of GHL10 domains** | **number of species** |
| Bacteroidetes  Bacteroidia  Sphingobacteriia  Cytophagia | 468  310  50  46 | 237  133  24  34 |
| Cyanobacteria  Nostocales  Oscillatoriophycideae  Synechococcales | 421  177  156  65 | 98  29  31  31 |
| Firmicutes  Clostridia  Bacilli | 260  130  106 | 198  109  75 |
| Actinobacteria  Streptomycetales  Micrococcales | 147  97  14 | 135  92  14 |
| Proteobacteria | 90 | 83 |
| Deinococcus-Thermus | 18 | 12 |
| Fusobacteria | 12 | 11 |
| Planctomycetes | 12 | 9 |
| Verrucomicrobia | 11 | 8 |
| Gemmatimonadetes | 11 | 5 |
| Tenericutes | 9 | 4 |
| Thermotogae | 8 | 6 |
| Ignavibacteriae | 5 | 2 |
| Spirochaetes | 4 | 3 |
| Chloroflexi | 4 | 3 |
| Chlorobi | 4 | 3 |
| Armatimonadetes | 4 | 2 |
| Deferribacteres | 4 | 1 |
| Acidobacteria | 3 | 2 |
| Haloplasmatales | 2 | 1 |
| Lentisphaerae | 1 | 1 |
| Candidatus Omnitrophica | 1 | 1 |
| Nitrospirae | 1 | 1 |
| Candidatus Cloacimonetes | 1 | 1 |
| **ARCHAEA** | | |
| **Phylum** | **number of GHL10 domains** | **number of species** |
| Archaea candidate phyla | 7 | 2 |
| Euryarchaeota | 1 | 1 |
| **EUKARYOTA** | | |
| **Phylum** | **number of GHL10 domains** | **number of species** |
| Metazoa | 14 | 5 |
| Fungi | 1 | 1 |
