# Supplementary figures and images for: Cwp19 Is a Novel Lytic Transglycosylase Involved in Stationary-Phase Autolysis Resulting in Toxin Release in Clostridium difficile
Source: mBio. 2018 Jun 12;9(3):e00648-18. doi: 10.1128/mBio.00648-18 (PMC6016235; doi:10.1128/mBio.00648-18)

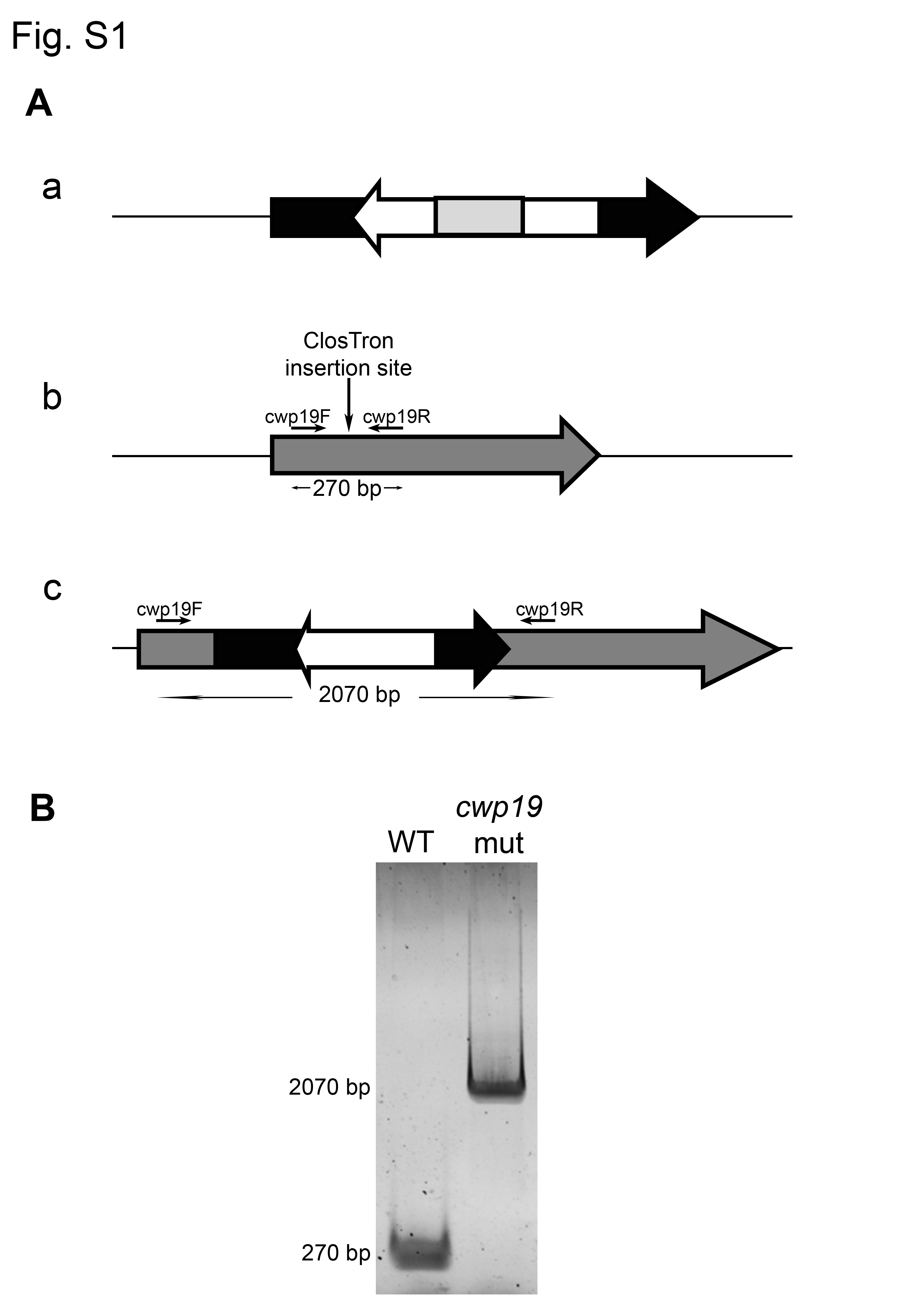

Supplement: FIG S1 [file mbo003183933sf1.tif]

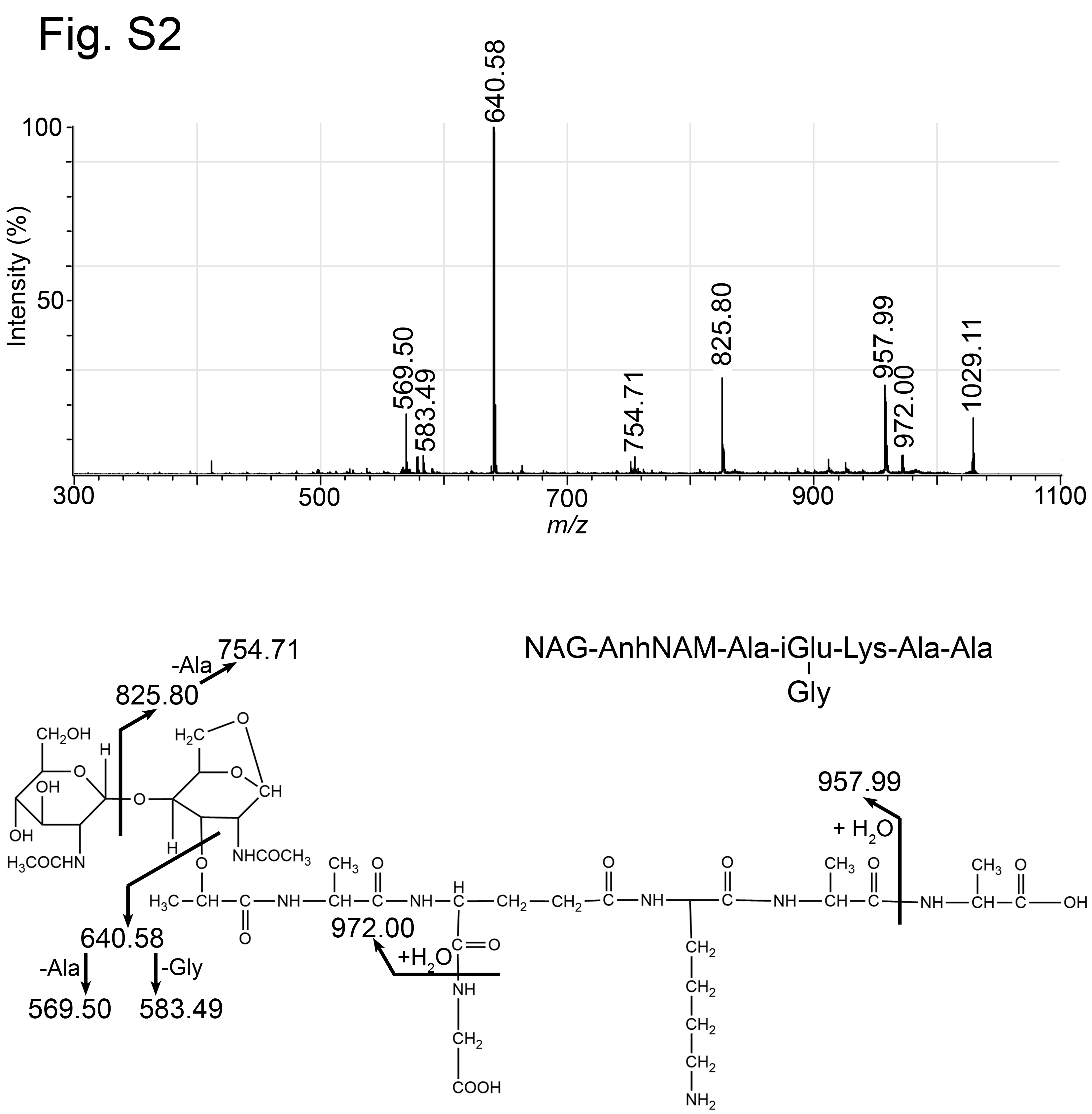

Supplement: FIG S2 [file mbo003183933sf2.tif]

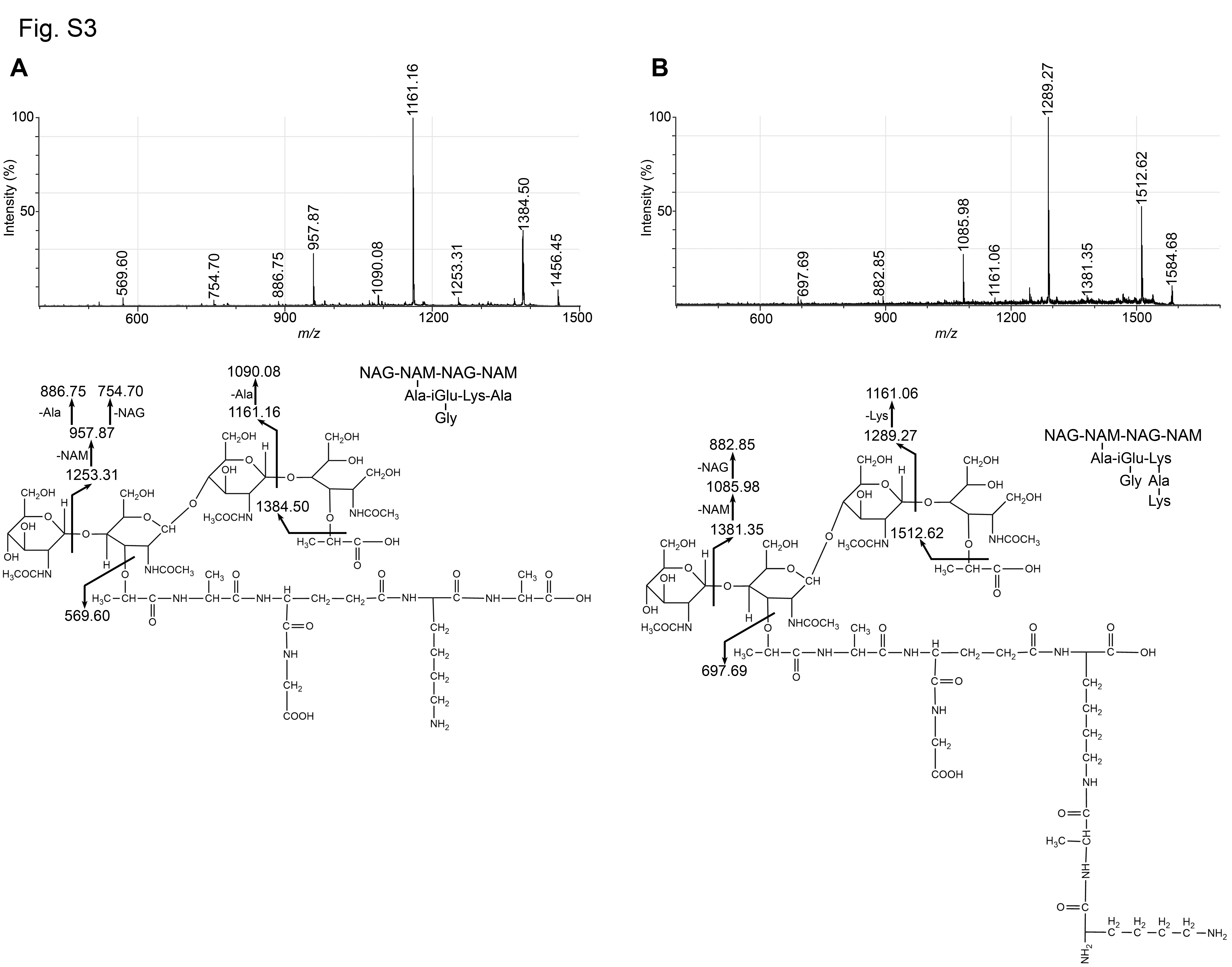

Supplement: FIG S3 [file mbo003183933sf3.tif]

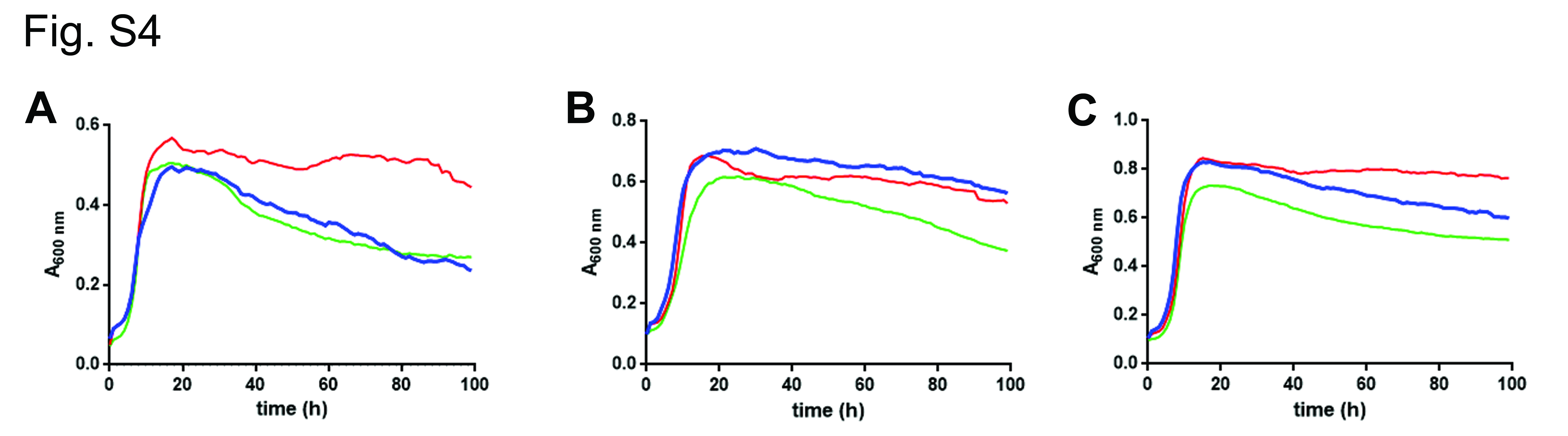

Supplement: FIG S4 [file mbo003183933sf4.tif]

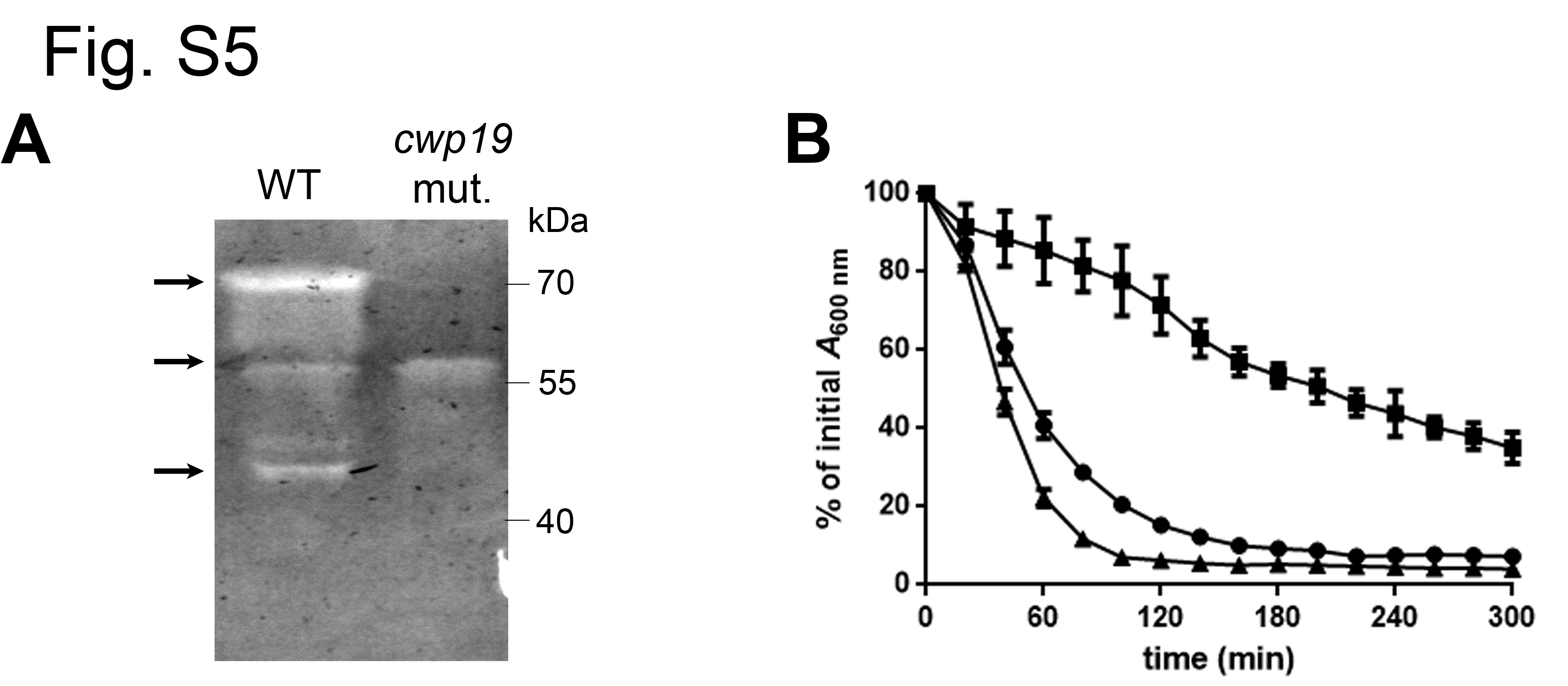

Supplement: FIG S5 [file mbo003183933sf5.tif]
