# Supplementary material for: Cwp19 Is a Novel Lytic Transglycosylase Involved in Stationary-Phase Autolysis Resulting in Toxin Release in Clostridium difficile
Source: mBio. 2018 Jun 12;9(3):e00648-18. doi: 10.1128/mBio.00648-18 (PMC6016235; doi:10.1128/mBio.00648-18)
Supplement: TABLE S3 [file mbo003183933st3.docx]

**Table S3.** Strains, plasmids and primers used in this study.

| **Strains** | **Relevant features** | **Reference or source** |
| --- | --- | --- |
| *C. difficile* |  |  |
| 630Δerm | Erm^S^ | ([1](#_ENREF_1)) |
| 630Δerm *cwp19::intron-erm* | Erm^R^ | This study |
| 630Δerm *cwp19::intron-erm* + pCOMP-*cwp19* | Erm^R^, Cm^R^ | This study |
| *E. coli* |  |  |
| TOP10 | F^-^ mcrA D(mrr-hsdRMS-mcrBC) f80lacZDM15 DlacX74 deoRrecA1 araD139 D(ara-leu)7697 galKrpsL(StrR) endA1 nupG | Invitrogen |
| HB101 (RP4) | supE44 aa14 galK2 lacY1 D(gpt-proA) 62 rpsL20 (Str^R^) xyl-5 mtl-1 recA13 D(mcrC-mrr) hsdS_B_(r_B_^-^m_B_^-^) RP4 | Laboratory stock |
| **Plasmids** | **Relevant features** | **Reference or source** |
| RP4 |  |  |
| pMTL007 | group II intron, ErmBtdRAM2 and ltrA ORF from pMTL20lacZTTErmBtdRAM2 Cm^R^ | ([2](#_ENREF_2)) |
| pMTL007::*cwp19*-588s | Cm^R^ | This study |
| pCOMP*19* | *cwp19* with its own promoter into pMTL007 | This study |
| pRPF185 | *E. coli*-*C. difficile* shuttle vector for protein expression. *P_tet_-gusA*, Tm^R^ | ([3](#_ENREF_3)) |
| pSUR19 | pRPF185 derivative carrying *P_tet_-cwp19* for inducible Cwp19 expression | This study |
| **Primers** | **Sequence (5’→3’)** | **Features** |
| Oligo dC-Race | GACCACGCGTATCGATGTCGACGGGGGGGGGGGGGGGGH | 5’ RACE PCR |
| cwp19 sp1 | CAGGGTCTTTACCTTGTGTAC | 5’ RACE PCR |
| cwp19 sp2 | GTCTTACTTGAACCACTGCTG | 5’ RACE PCR |
| cwp19 sp3 | CTGTAGATATCCAAGCAGCTC | 5’ RACE PCR |
| EBSuniversel | CGAAATTAGAAACTTGCGTTCAGTAAAC | Mutagenesis |
| IBS cwp19 | AAAAAAGCTTATAATTATCCTTAGCAGACGTAGTTGTGCGCCCAGATAGGGTG | Mutagenesis *cwp19* pt588 |
| EBS1d cwp19 | CAGATTGTACAAATGTGGTGATAACAGATAAGTCGTAGTTCATAACTTACCTTTCTTTGT | Mutagenesis *cwp19* pt588 |
| EBS2 cwp19 | TGAACGCAAGTTTCTAATTTCGATTTCTGCTCGATAGAGGAAAGTGTCT | Mutagenesis *cwp19* pt588 |
| cwp19 F | CTACGACCCAGGTTTACC | Mutagenesis confirmation |
| cwp19 R | CATATACCTGCTGGGCTTAC | Mutagenesis confirmation |
| ErmRAM F | ACGCGTTATATTGATAAAAATAATAATAGTGGG | Mutagenesis confirmation |
| ErmRAM R | ACGCGTGCGACTCATAGAATTATTTCCTCCCG | Mutagenesis confirmation |
| pMTLseq-F | GGGATCCTCTAGAGTCG | intron retargeted sequencing |
| pMTLseq-R | CAGATTCTCGGCATCGC | intron retargeted sequencing |
| cwp19 compF | TTATGCTCGAGGACTTATAACTGCTACACGTGTTTTAC | *cwp19* complementation |
| cwp19 compR | TTTTACGATCGCTTTTTTATTACTTAACTAAGTTTAAGAAAGTG | *cwp19* complementation |
| pMTLcompF | GACCATGATTACGAATTCGAGC | Complementation confirmation |
| pMTLcompR | GCTGCTGCATCTCTTCGC | Complementation confirmation |
| DNA polIII F | TCCATCTATTGCAGGGTGGT | qRT-PCR |
| DNA polIII R | CCCAACTCTTCGCTAAGCAC | qRT-PCR |
| cwp19RT-F | ATATGACACAGCAGTAGCAATAAG | qRT-PCR |
| cwp19RT-R | AGCAAGTGGTGTTGATGTTATAC | qRT-PCR |
| cwp19 surF | AGCACTCGAGTTAAGGGGGATGAAATGAA | Cwp19 overexpression |
| cwp19 surR | AGCAGGATCCTTAATGGTGGTGGTGATGATGCTTAACTAAGTTTAAGAAAGTGTTTAATG | Cwp19 overexpression |

**REFERENCES**

1. **Hussain HA, Roberts AP, Mullany P.** 2005. Generation of an erythromycin-sensitive derivative of *Clostridium difficile* strain 630 (630Deltaerm) and demonstration that the conjugative transposon Tn916DeltaE enters the genome of this strain at multiple sites. J Med Microbiol **54:**137-141.

2. **Heap JT, Pennington OJ, Cartman ST, Carter GP, Minton NP.** 2007. The ClosTron: a universal gene knock-out system for the genus *Clostridium*. J Microbiol Methods **70:**452-464.

3. **Fagan RP, Fairweather NF.** 2011. *Clostridium difficile* has two parallel and essential Sec secretion systems. J Biol Chem.
